# A Comparison of Spatial Analysis Methods for the Construction of Topographic Maps of Retinal Cell Density

**DOI:** 10.1371/journal.pone.0093485

**Published:** 2014-04-18

**Authors:** Eduardo Garza-Gisholt, Jan M. Hemmi, Nathan S. Hart, Shaun P. Collin

**Affiliations:** School of Animal Biology and The UWA Oceans Institute, The University of Western Australia, Crawley, Western Australia, Australia; Federal University of Rio de Janeiro, Brazil

## Abstract

Topographic maps that illustrate variations in the density of different neuronal sub-types across the retina are valuable tools for understanding the adaptive significance of retinal specialisations in different species of vertebrates. To date, such maps have been created from raw count data that have been subjected to only limited analysis (linear interpolation) and, in many cases, have been presented as iso-density contour maps with contour lines that have been smoothed ‘by eye’. With the use of stereological approach to count neuronal distribution, a more rigorous approach to analysing the count data is warranted and potentially provides a more accurate representation of the neuron distribution pattern. Moreover, a formal spatial analysis of retinal topography permits a more robust comparison of topographic maps within and between species. In this paper, we present a new R-script for analysing the topography of retinal neurons and compare methods of interpolating and smoothing count data for the construction of topographic maps. We compare four methods for spatial analysis of cell count data: Akima interpolation, thin plate spline interpolation, thin plate spline smoothing and Gaussian kernel smoothing. The use of interpolation ‘respects’ the observed data and simply calculates the intermediate values required to create iso-density contour maps. Interpolation preserves more of the data but, consequently includes outliers, sampling errors and/or other experimental artefacts. In contrast, smoothing the data reduces the ‘noise’ caused by artefacts and permits a clearer representation of the dominant, ‘real’ distribution. This is particularly useful where cell density gradients are shallow and small variations in local density may dramatically influence the perceived spatial pattern of neuronal topography. The thin plate spline and the Gaussian kernel methods both produce similar retinal topography maps but the smoothing parameters used may affect the outcome.

## Introduction

Topographic density maps are an informative and intuitive way of representing graphically the distribution of different types of cells in the retina. They are particularly useful for identifying retinal specialisations that may reflect an animal's visual ecology and/or phylogeny. Topographic maps of retinal ganglion cell and photoreceptor distributions also provide crucial information about the limits of visual resolution and the degree of signal convergence (i.e. sensitivity). As a result, hundreds of maps have been produced in the last decades for a range of vertebrate and many invertebrate species [1], [2]. All species of vertebrates studied to date have some form of retinal specialization, rather than a uniform distribution of neurons, and each retinal specialization varies greatly in shape, size and number depending on the ecology and the phylogeny of the species. Retinal specialisations are usually defined as a localised area of increased cell density that affords increased spatial sampling of a specific region of an animal's visual field [3].

A commonly observed retinal specialization is that of a radially symmetric gradient of increasing cell density. When the central zone of highest cell density (the ‘*area*’) is located in central retina, this specialisation is called an *area centralis* and when it is located in dorsal retina, it is called an *area dorsalis*, and so on. More than one retinal *area* may be present, as is common in many fishes and birds, which have both an *area centralis* and an *area temporalis* [1]. Another form of specialization is an elongated band of high cell density that extends across a significant portion of the retina and is called a ‘visual streak’. The primary axis of the streak may be oriented vertically or horizontally or even curved to form a ‘dorsal arch’ [4]–[6]. This type of specialisation provides high acuity vision across an extended portion of the visual scene without the need for extensive scanning eye movements [7], [8].

In some species of vertebrates, the zone of highest cell density within an *area* or a visual streak is characterised by an indentation of the retinal layers to form a pit or ‘fovea’ in the retina. Usually, the cell bodies of all but the photoreceptors are displaced to the sides of the foveal pit to provide unimpeded access of light to the rods and/or cones to mediate high acuity vision. The shape of the fovea may also provide some optical magnification of the image due to the difference in the refractive indices between the vitreous and the retinal tissue lining the fovea [9]–[11]. In some deep-sea species, the fovea in each eye may distort the image of bioluminescent light flashes enough to mediate some form of depth perception, while providing enhanced sampling with large numbers of tightly-packed rod photoreceptors [12]–[14].

These retinal specializations can be found in various combinations and forms and show a marked interspecific variability. The position, shape, retinal coverage and centro-peripheral density gradient of each acute zone can be used to infer important information about the visual ecology of each species and their visual environment. Given the importance of topographic maps of retinal neurons for understanding the visual ecology of a species, it is critical that these distribution maps are as accurate as possible and that artefacts introduced by the techniques used, such as the preparation of the retinal wholemount [15], [16] and the staining and visualisation of the retinal neurons, are minimized as much as possible [17].

Traditionally, topography maps have been constructed from localised cell counts using a simple pairwise interpolation of adjacent points on the retina to calculate iso-density values that can be joined by contour lines [4], [18]–[20]. However, this method does not consider or interpolate the distances between all points, but only the distances of adjacent points where the iso-density lines are constructed. The method also relies on the researcher's ability to discriminate the best interval of the contour lines, which can be subjective. More recent retinal studies use a variation of this method, where authors tend to employ a “smooth by hand or by eye” method that only means that some points might be arbitrarily removed or that the line might be “curved” to give a better visual appearance [21]–[24].

Some authors have used computer methods to analyse the iso-density maps but, in some cases, the methodology is not clear and reproducible. The method used to determine the contour lines is often not stated so it is not possible to determine if any information is lost because of any type of smoothing or whether maps can be compared if they use different kinds of spatial analyses. Some authors use a digital planimeter to digitize the iso-density contours and measure regions [25], [26]. Do-Nascimento *et al.* [26] even include a different type of map, indicating differences in cell density with different sized dots [26]. Mass and Supin [27]–[29] have published a diversity of maps, where the contours were manipulated by computer and the number of cells were averaged (smoothed) in blocks of 3×3 samples. They have also devised an original method to transform the map into a continuous spherical representation [27]–.

Stone and Halasz [30] used a dotted map to represent the magnitude of changes in cell density and a grey scale gradient with discrete steps generated using a computer-based paradigm in the elephant retina [30]. Fischer and Kirby [31] used the Golden software package (Surfer) to calculate the iso-density lines of the topographic maps in *Anubis* baboons [31]. More recently, the geographic information system (GIS) has been used to analyse cell topography in retinas, where ArcGIS (Esri) systems like ArcView and ArcMap have been used to analyse retinal cell density gradients in birds [32]–[34]. However, even with the use of powerful software packages (i.e. Systat [35] and graphical programs like DeltaGraph [36]–[38], the methodology used in most studies is insufficient to assess the accuracy of the spatial analysis although Hemmi and Grünert [39] used a slightly smoothed thin plate spline interpolation [39] and Coimbra *et al* [6], [40] specified the use of a spline interpolation in penguins [6] and giraffe [41].

The statistical language ‘R’ is an inexpensive open access program [42] providing flexibility, a range of mathematical functions and online help including forums for experts to help users with specific questions. Spatial analysis using the R program has been applied in diverse fields. For example, the *spatstat* package was used to analyse the relationship between the population of tuna and the oceanographic conditions [43] was and the insect distribution changes as a result of habitat loss [44]. R has recently been used to develop an extensive range of spatial analysis models for the examination of spatial patterns in animal disease [45]. There are more than 30 different methods of interpolation and smoothing models that could create maps in R, making it a powerful tool for spatial analysis [45]–[47].

A comparison between different algorithms is necessary to identify which model can be adapted more easily to faithfully represent the topographic distribution of retinal cells. These algorithms come in two basic forms. Firstly, interpolation models interpolate the data to fill gaps and provide a sampling base for graphical representation. The observed data is not modified by the function so the resulting curve or surface will cross the sample locations at the exact observed value [46]. In contrast, spatial analysis that smoothes the data, uses the spatial position and the magnitude between the observed points to obtain a spatial function to calculate the cell densities in the different areas of the retina. The use of smoothing analysis removes outliers from the sampling and reduces local variation caused by potential artefacts [48], [49].

In this study, we present results comparing maps generated by different algorithms in R, and compare the resulting maps with previously published, hand-generated iso-density maps showing a range of different retinal specializations. The algorithms provide an objective, reliable and improved representation of the topographic distributions of cells in the retina. The ability to automate map generation also saves significant time compared to traditional methods.

## Methods

We analysed six different, previously published, topographic maps sampling retinal ganglion cell populations that were Nissl stained and counted under a compound microscope following the technique of Collin and Pettigrew [4], [20]. The topographic maps analysed were the reef fish: *Cephalopholis miniatus* with a temporal *area*, *Amplyglyphidodon curacao* with multiple areas, *Parapercis cylindrica* with a horizontal streak and two areas [20]; *Choerodon albigena* with a horizontal streak and a dorso-temporal *area*, *Gymnocranius bitorquatus* with a dorsal *area* [4] and a deep sea fish *Conocara murrayi* with a fovea [5]. The original papers can be found attached in File S5 (Collin and Pettigrew 1988.pdf); File S6 (Collin and Pettigrew 1988b.pdf); and File S7 (Collin and Partridge 1996.pdf). The original iso-density contour maps were based on cell counts that were interpolated by hand and were obtained with the permission of the authors. An additional iso-density contour map of ganglion cell distribution in the retina of the pacific spookfish, *Rhinochimaera pacifica*, illustrating the horizontal streak was analysed using the stereological method using *StereoInvestigator* software (Microbrightfield, USA) to sample the retina in an unbiased and automated way [33] (“File S1 retina.xml”). Therefore, by including data collected and analysed using two different methods, it will be possible to objectively compare the neuronal distribution patterns generated by this new technique and how this may affect their interpretation.

Input data was generated either by digitising hand drawn counting maps or by reading in output files from the *StereoInvestigator* program. Original counting maps that show the position of each cell count and the calibrated calculations of cells per square millimetre at each retinal loci were scanned and digitized using Adobe Illustrator CS5 (San Jose, Calif., USA). The outline of the retina, the position of the optic nerve head and any retinal artefacts were all digitised as *polyline* objects. To create a *polyline* object, we selected the *pen tool* and clicked on each point of the outline. The positions of the observations were placed like text inside the created outline. A copy of the file was saved as a scalable vectors graphics (.svg) file that can be assessed in a text editor program such as WordPad to identify the different sections of the file that would be extracted (“File S3 A curacao 23.svg”). The outline of the retina and the outlines representing the optic nerce and other retinal artifacts should be of the type “*polyline*”. If they are saved as “*path*” then it means they are not straight lines and include curves. To convert a *path* to a *polyline*; it is possible to select the *path*, go to *Object>Path>Simplify* and select the straight lines option (“File S4 One cell map V8 svg version.R”). Alternatively, *StereoInvestigator* can be used to generate the cell counts. The data needs to be saved in extensible markup language (.xml) format. The xml file includes the x and y coordinate positions and the type of marker for each object. For the spatial analysis, the script counts the cells and obtains the average position of the x and y coordinates for each site analysed. Additional modifications were necessary to obtain the correct value and the position of each site using the *plyr* [50] and *stringr* packages in R [51]. More details can be found in the electronic version of the script (“File S2 One cell map V8.R”).

The data obtained was analysed spatially using R. Four different algorithms were used to spatially analyse the data, and construct iso-density maps: 1) an *akima* interpolation (*Akima package*), 2) a thin plate spline interpolation (*fields* package), 3) a thin plate spline (Tps) (*fields* package), and 4) a Gaussian kernel smoother (Gks) (*spatstat* package). The first two algorithms interpolate the data without modifying the observed values while Tps and Gks smooth the observed data.

The two interpolation models respect the original observations and only fill in empty spaces using different algorithms. Akima bivariate interpolation is a fifth degree polynomial function in the x-y plane, which creates a series of triangles to estimate partial derivatives. This method is useful when some areas of the retina were not sampled due to damage, because it considers irregular-spaced samples [52], [53]. The second model uses a spline to obtain the interpolation. The thin plate spline is a geometric function that ‘bends’ the spatial arrangement to adjust to the values of the points. The smoothness of the thin plate spline can be adjusted in two ways: firstly, the thin plate spline uses a roughness penalty that is known as the penalized sum of squares, the strength of which can be adjusted through lambda (λ) and works as a smoothing parameter. If the smoothness parameter lambda is set to 0 then the data is not deformed and the original values are respected thereby creating a cubic interpolation [54]. The second way to adjust the smoothness is with the degrees of freedom that are the effective number of parameters used to fit the surface model. A lower number of degrees of freedom will increase the smoothing of the data [49], [55]. For the present comparison, we used one third of the number of observations for the over-smoothed model and two thirds of the number of the observations for the under-smoothed model. Like the Tps, the Gaussian kernel smoother (Gks), smoothes the data locally and can adjust the observed data depending on neighbouring observations. The Gaussian kernel smoother, creates a series of Gaussian distributions to fit the spatial data. The smoothness can be controlled by the smoothing kernel bandwidth that can be modified with the use of the sigma value close to the distance between observations [48], [56].

The resulting functions for each model were used to calculate the required points in the maps to construct the iso-density contours. A grid of 200 µm of resolution was created to input in the function to predict the values in each point of the grid. The iso-density contour levels were set to the original values presented in the published maps and in some cases the number of levels was modified to compare the effects of using different iso-density lines. For each of the smoothing models, two parameters were compared giving an under-smoothed and an over-smoothed iso-density map. In the results of the paper, we present only a limited number of comparisons for each retina to illustrate different scenarios and enable a decision to be made as to the most appropriate type of analysis.

The foveal specialization (a very sharp peak of high cell density) represents a special case that required a different approach because of the magnitude of the cell density gradient. The interpolation model and the smoothed model were compared to a third approach. A hybrid of two models was used, where the foveal region was analysed as a subset of the data and isolated from the rest of the retina. The cell distribution in the fovea was analysed with a Tps interpolation and the Tps smoothed model was used to analyse cell distribution in the retinal periphery; the data was then superimposed and combined in a single topographic map.

To compare the models, we plotted the density function for all models [57]. This approach cannot however be used to compare the models to the original dataset, as the data is not normally evenly sampled across space, leading to a distortion in the density distribution. An alternative way to present the density distribution is to plot the cumulative distribution function. For retinal analysis, this is informative, because it allows one to easily read out how many cells a retina contains at different cell densities, which helps in comparing retinae across species. One way to analyse the change of the data from the smoother is to use a residual analysis. Residual values were obtained calculating the percentage of difference between the observed data and the modelled data. These residuals should be distributed randomly and with equal variance across the retina. The percentage range gives an indication of how strongly the observed data was modified. Mapping the residuals in the same way we mapped the original density values, provides a graphical tool to check whether or not the data smoothing has affected the shape of the density distribution. Strong spatial structure in these plots indicates that the shape of the density distribution has been modified by the smoothing algorithm. Probably the most informative and direct way to assess the model fit (and at the same time reduce the dimensions of the spatial interpretation) is to plot the values along a transect line, i.e. horizontal and vertical transects across the maps, showing measured and estimated data points [58].

R is a platform independent open source program (i.e. Windows, Mac, and Linux) that is available from the Comprehensive R Archive Network (CRAN), (http://cran.r-project.org) [42]. We used the R Studio that is an Integrated Development Environment that helps novice users making R more user-friendly (http://www.rstudio.com) [59]. The R script for the extraction and analysis of the data presented here is included as electronic supplemental material and can be used and distributed freely as long as the authors, the creators of R and the different packages used in the script are all cited appropriately. There are two versions of the script; one version extracts the data in the form of an Extensible Markup Language (.xml) file that is suitable for exported files from *StereoInvestigator* and the other version extracts the values from a Scalable Vectors Graphic (.svg) file that can be obtained from *Adobe Illustrator*, *Corel Draw*, *Inkspace* and other vector graphics software packages. The respective R scripts with an example file for a xml format file (“*File S1 retina.xml*” that runs with the “*File S2 One cell map V8.R*” script) and a svg format file (“*File S3 A curacao 23.svg*” that runs with the “File S4 *One cell map V8 svg version.R*” script) retinas can be found in the supplementary electronic materials.

## Results

### Choosing between different models

**Figure 1:**
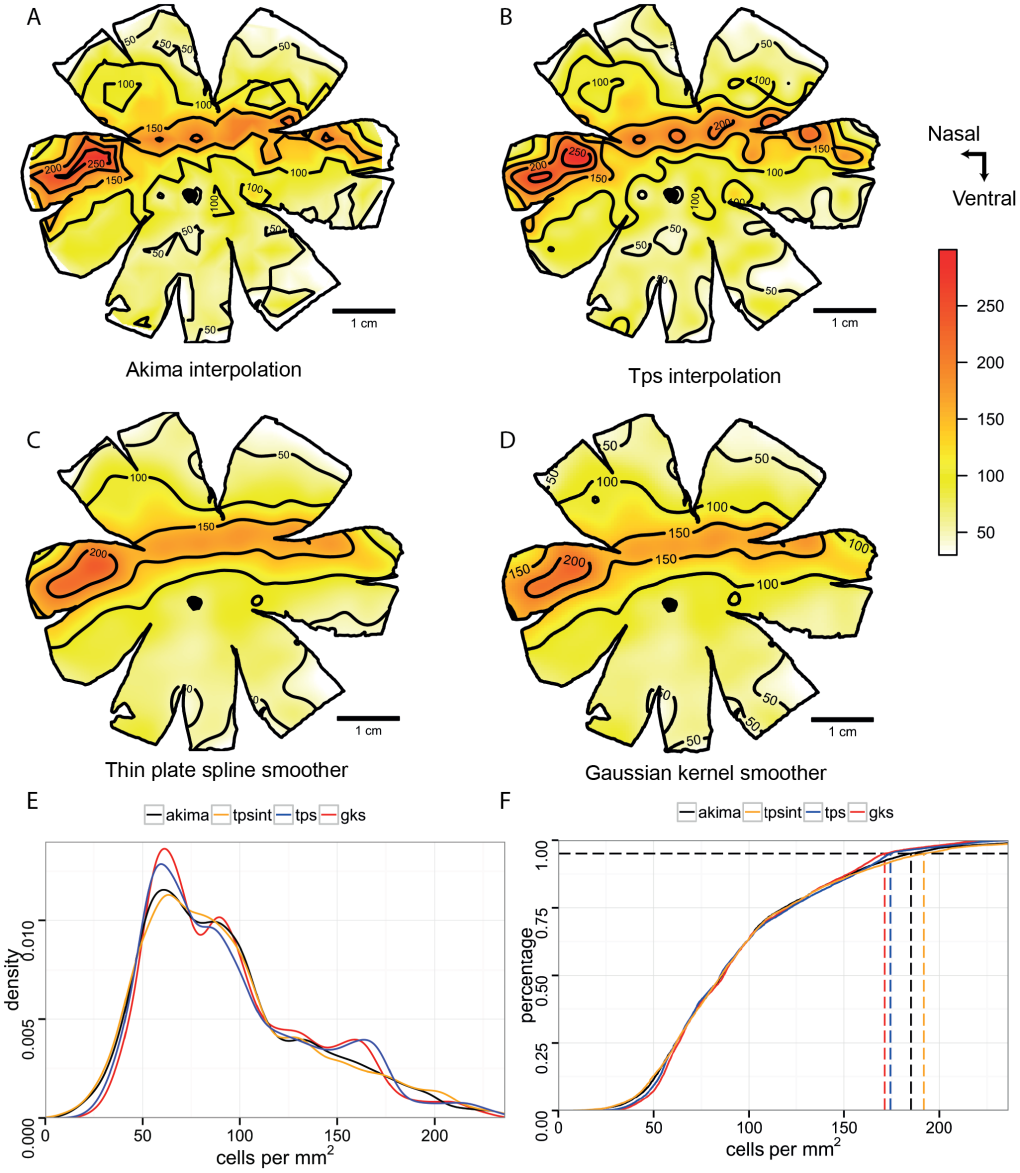
Topographic maps of the ganglion cells in the retina of *Rhinochimaera pacifica*. Topographic maps of the ganglion cells in the retina of the Pacific spookfish, *Rhinochimaera pacifica*. A. Akima interpolation. B. Thin plate spline interpolation. C. Thin plate spline smoother. D. Gaussian kernel smoother. E. Distribution density curves of the 4 models. F. Cumulative distribution functions for the four models; dash lines denote cell density higher of the 95% of the area of the retina. All the densities are in cells mm^−2^. The maps were predicted with a distance of 200 µmbetween points.

All four iso-density contour maps constructed using data obtained from the Pacific spookfish, *Rhinochimaera pacifica* using the four different analysis methods reveal a pronounced horizontal streak. These results are novel and the biological significance of the observed topographic distribution are discussed elsewhere (Garza-Gisholt *et al.*, in prep). The interpolation models show more local variation in the retina (noise) with discontinuous iso-density areas; while the smoothed models show a similar overall topographic pattern but with less local variation. Comparing the two interpolation models, the main difference between the two interpolation algorithms is that the *Akima* interpolation (Figure 1A) uses a triangular calculation and so the nodes of the lines are sharp, while spline interpolation (Figure 1B) shows curved lines because the algorithm uses a cubic function to interpolate the data. The two smoothing algorithms give almost identical results. The only difference is that the output of the Gaussian kernel model (Figure 1D) gives an image from the calculated points, while thin plate spline model (Figure 1C) gives a series of equations that allows densities to be calculated for any point inside the retina. The comparison between the density lines of the models shows that the two interpolations and the two smoothing models are similar. The peak of the distribution curve reveals what cell density value is most abundant in the retina and does not represent the peak density; when the data is smoothed, the peak tends to narrow (Figure 1E). The cumulative distribution function is more intuitive to assess how broad or narrow the retinal area is. If we look at the cut-off density that contains the top 5% of density values, the shallower the curve at the top, the faster is the drop in density away from the peak. The cut-off value is higher in the spline model than the *Akima* interpolation model, while the smoothed models both have a lower value (Figure 1F), indicating that the area around the peak density has been slightly flattened out by the smoothing procedure.

### Choosing different smoothing parameters

We assessed all the retinas using the four models and concluded that the interpolation models are very similar, but the thin plate spline model has the advantage of a smoother appearance. The smoothed models are different to the interpolation models but show both the same general patterns. The Tps has the flexibility to manipulate the smoothness from a linear interpolation with no smoothness to an over smoothed analysis. Therefore, for the next six retinas, we present the figures for the thin plate spline (Tps) model only.

**Figure 2:**
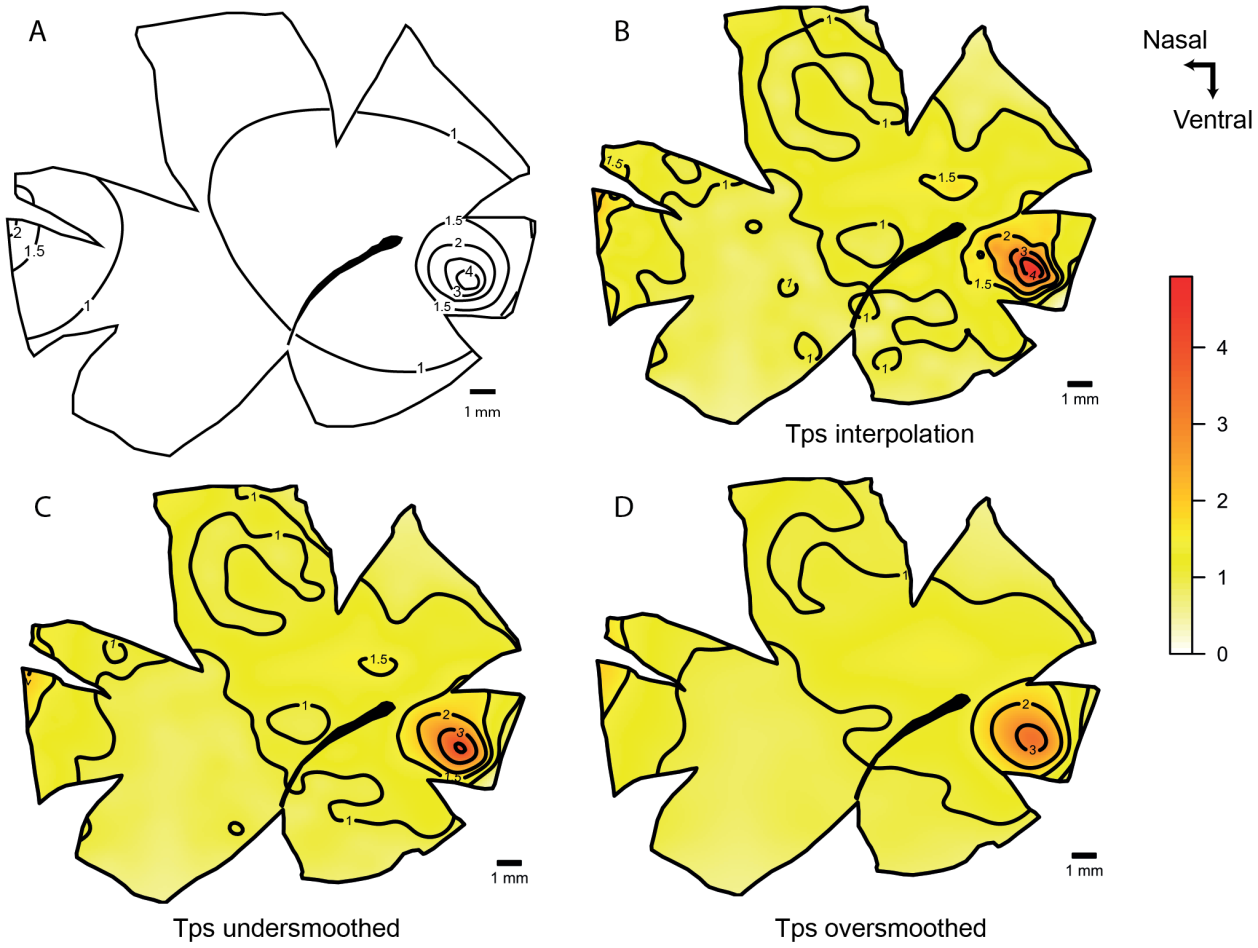
Topographic maps of the ganglion cells showing *area temporalis* in *Cephalopholis miniatus*. Topographic maps of the ganglion cells in the retina of the coral cod, *Cephalopholis miniatus* that show an *area temporalis* specialization. A. Modified map from Collin and Pettigrew, 1988a. B. Thin plate spline interpolation using a lambda value of 0. C. Thin plate spline under-smoothed with the 66% of count numbers for calculated degrees of freedom. D. Thin plate spline over-smoothed using the 33% of count numbers for calculated degrees of freedom. All the densities are ×10^4^ cells mm^−2^. The maps were predicted with a distance of 200 µm between points.

**Figure 3:**
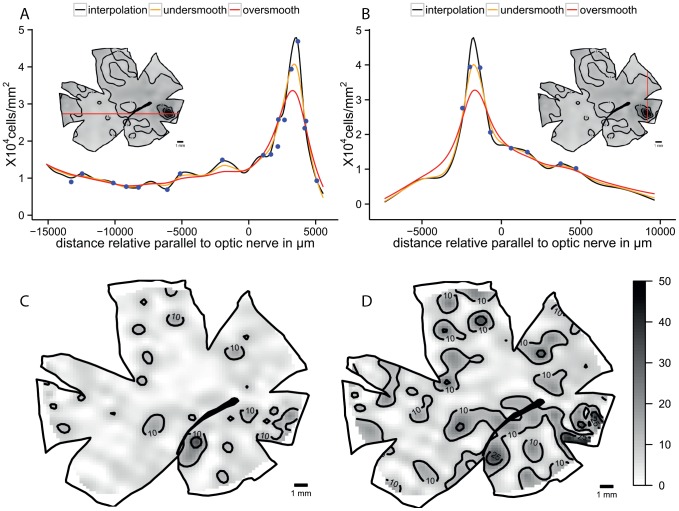
Diagnostic analysis of the maps for *Cephalopholis miniatus*. Horizontal and vertical transects, and residual analysis of the two levels of smoothness for the coral cod, *Cephalopholis miniatus*. A. Horizontal transect crossing the highest density area, the dots represent observed points that are in the transect or at a distance not higher than 0.5 mm of the transect line. B. Vertical transect crossing the highest density area, the dots represent observed points that are in the transect or at a distance not higher than 0.5 mm of the transect line. C. Residual map for the under-smoothed model showing percentage of variation between the calculated data and the observed data, the contour lines represent the areas that varied more than 10% and 25%. D. Residual map for the over-smoothed model showing percentage of variation between the calculated data and the observed data, the contour lines represent the areas that varied more than 10% and 25%.

In the coral cod, *Cephalopholis miniatus*, an *area* is present in the ventro-temporal retina. The original map shows little variation in the iso-density contours, which emanate from the regions of highest cell density (predominantly spaced in 10,000 cells mm^−2^ increments). In comparison, spline interpolation shows higher local variation that is underestimated in the traditional ‘by hand’ or ‘by eye’ interpolation model. Comparing the spline interpolation, an under-smoothed model and an over-smoothed model, the same *area* is present in all three maps. There is also a small *area* in the nasal retina present in the original map but the cell density is lower and it is not evident in the interpolation or the smoothing maps (Figure 2). The two transects crossing the retinal specialization, show higher variation in the curves in the low cell densities that is reduced with the smoothed models. The high density peak in the smoothed models is reduced in comparison to the observed models; in a higher degree in the over smoothed model. The residual maps show more variation in the ventral and temporal areas, i.e. the variation from the over-smoothed model is higher showing a similar pattern in the residuals of the specialization. The residual values for the under-smoothed model are lower and the area of more than 25% change from the observed data is very small compared to the over-smoothed model (Figure 3).

**Figure 4:**
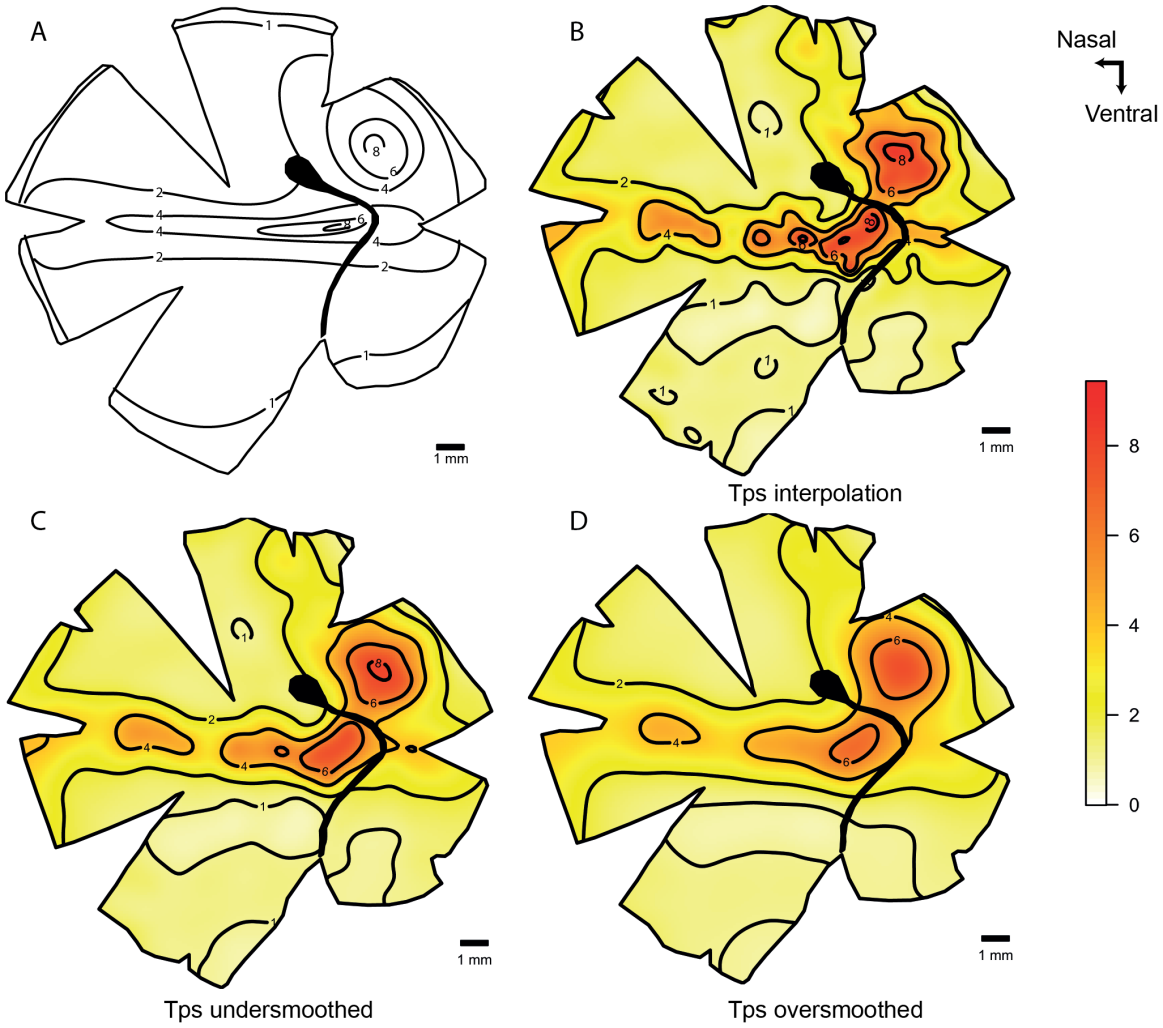
Topographic maps of the ganglion cells showing horizontal streak and *area* in *Choerodon albigena*. Topographic maps of the ganglion cells in the retina of the blue tusk fish, *Choerodon albigena*, that show a horizontal streak with a dorso-temporal *area* specialization. A. Modified map from Collin and Pettigrew, 1988b B. Thin plate spline interpolation using a lambda value of 0. C. Thin plate spline under-smoothed with the 66% of count numbers for calculated degrees of freedom. D. Thin plate spline over-smoothed using the 33% of count numbers for calculated degrees of freedom. All the densities are ×10^4^ cells mm^−2^.

**Figure 5:**
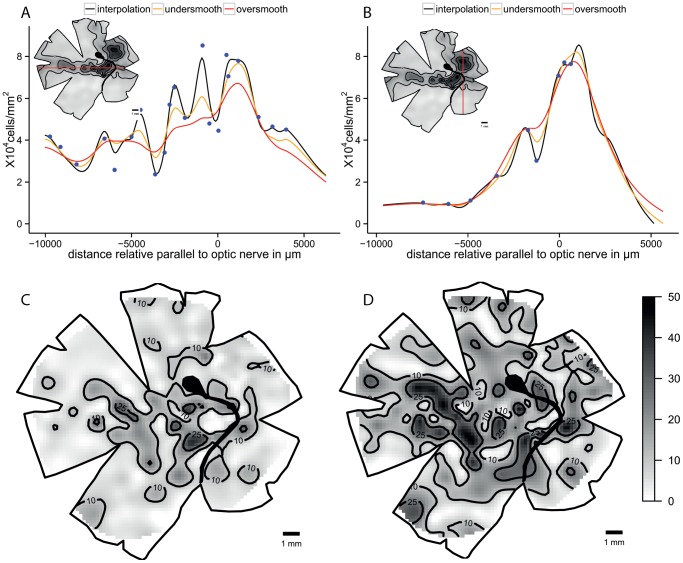
Diagnostic analysis of the maps for *Choerodon albigena*. Horizontal and vertical transects, and residual analysis of the two levels of smoothness for the blue tusk fish, *Choerodon albigena*. A. Horizontal transect crossing the highest density area, the dots represent observed points that are in the transect or at a distance not higher than 0.5 mm of the transect line. B. Vertical transect crossing the highest density area, the dots represent observed points that are in the transect or at a distance not higher than 0.5 mm of the transect line. C. Residual map for the under-smoothed model showing percentage of variation between the calculated data and the observed data, the contour lines represent the areas that varied more than 10% and 25%. D. Residual map for the over-smoothed model showing percentage of variation between the calculated data and the observed data, the contour lines represent the areas that varied more than 10% and 25%.

The blue tusk fish, *Choerodon albigena*, presents a horizontal streak combined with an *area centralis* in the dorso-temporal retina. The linear interpolation shows the same specialization but the horizontal streak values of 40,000 cells mm^−2^ are discontinuous. The smoothed models show a similar pattern with a horizontal streak of more than 20,000 cells mm^−2^ that extends to the dorso-temporal retina. Additionally there is a dorso-temporal *area* with a peak of 80,000 cells mm^−2^ in the under-smoothed model that is not present in the over-smoothed model but the lower centro-temporal *area* with a peak of 60,000 cells mm^−2^ is present in both models (Figure 4). The horizontal transect shows a high local variability in the streak affecting the continuity of the iso-density lines (Figure 4A). The high variability is reduced with the over-smoothing but the peak is lower than the interpolation line. The interpolation model shows that it is not a constant specialization and it has a lot of local variation. Along the vertical transect the observed cell densities are closely followed by the models and the smoothed models do not change the peak dramatically. The residual analysis shows a higher variation in the centre of the retina and the magnitude is higher but with no specific pattern revealed in the over-smoothed model (Figure 5).

### Analysing different specializations

The under-smoothed version of the Tps, using two thirds of the number of observations for the degrees of freedom, is more appropriate to analyse the retinas because it maintains the peak values at a level closer to the observed values. The local variation is higher than the over-smoothed model but it is generally preferable to keep it as the variation might contain useful information and reflect real biological variation rather than sampling noise. Therefore, the results of the three species presented below are analysed using the Tps interpolation and the under-smoothed Tps models.

**Figure 6:**
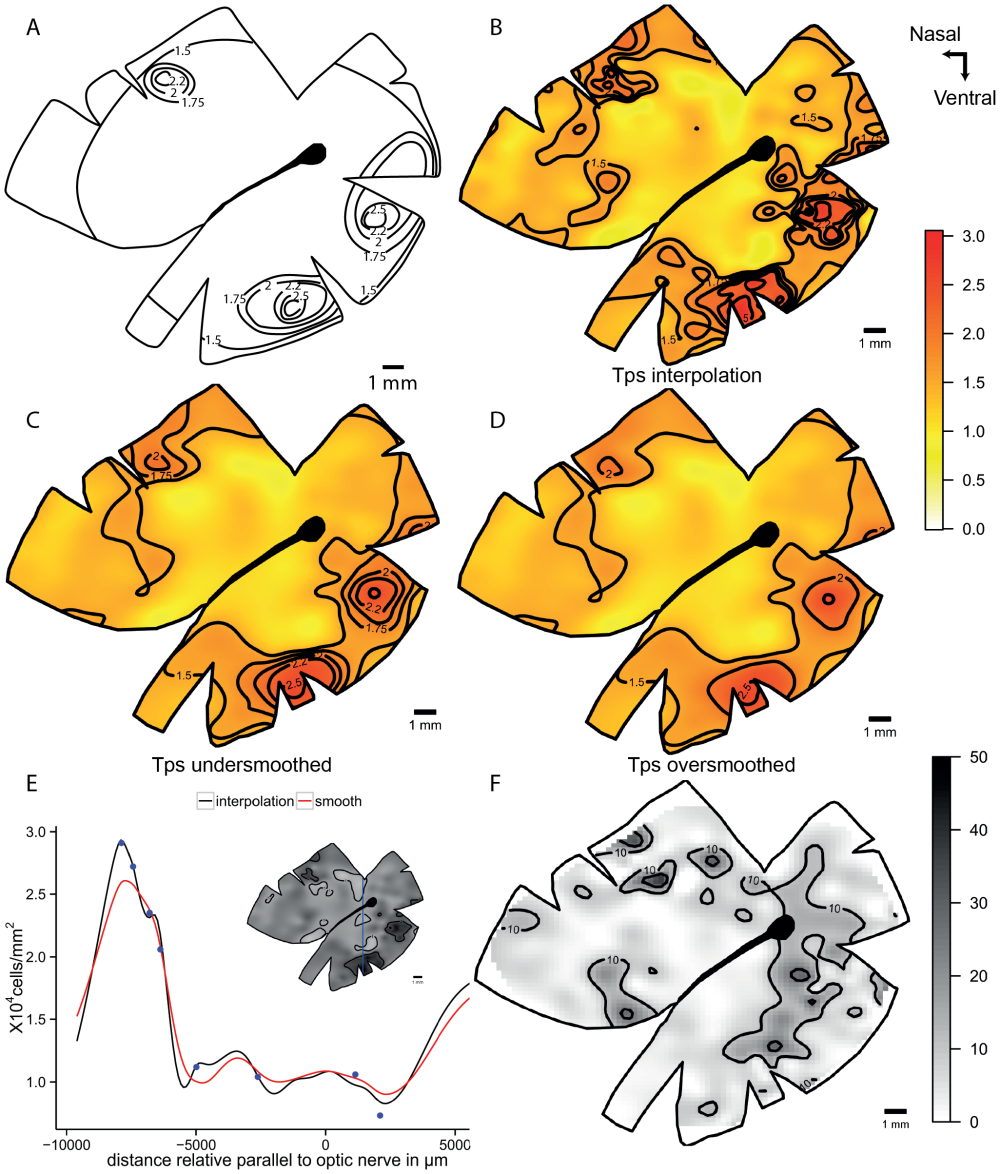
Topographic maps of the ganglion cells showing multiple *areae* in *Amblyglyphidodon curacao*. Topographic maps of the ganglion cells in the retina of the staghorn damselfish, *Amblyglyphidodon curacao*, that show multiple *areae* specializations. A. Modified map from Collin and Pettigrew, 1988a. B. Thin plate spline interpolation using a lambda value of 0. C. Thin plate spline under-smoothed map with the original isodensity contour levels. D. Thin plate spline under-smoothed map with lower number of isodensity contour levels. E. Horizontal transect crossing the highest density area, the dots represent observed data in no more than 0.5 mm to the transect line. F. Residual map for the over-smoothed model showing percentage of variation between the calculated data and the observed data, the contour lines represent the areas that varied more than 10% and 25%. All the densities are ×10^4^ cells mm^−2^.

The original map of the staghorn damselfish, *Amblyglyphidodon curacao*, shows a central plateau of more than 15,000 cells mm^−2^ where three specializations are present in the dorsal, temporal and ventral areas. In contrast, the Tps model shows that the ventral *area* and the temporal *area* are evident with the contour interval of 25,000 cells mm^−2^ in the periphery of the retina and a smaller dorsal *area* of 20,000 cells mm^−2^. The lowest density areas of the retina are in the central retina showing values of less than 15,000 cells mm^−2^. Only plotting the contour lines of 15,000, 20,000 and 25,000 cells mm^−2^ shows the same colour pattern but the presence of the specialization is clearer. The vertical transect running through the ventral specialization shows that the smoothed model follows the same pattern as the interpolated model but there is a reduction in noise in the lower density area. The residual map shows that the variation of the calculated points doesn't follow a specific pattern and is not affecting the specialization (Figure 6).

**Figure 7:**
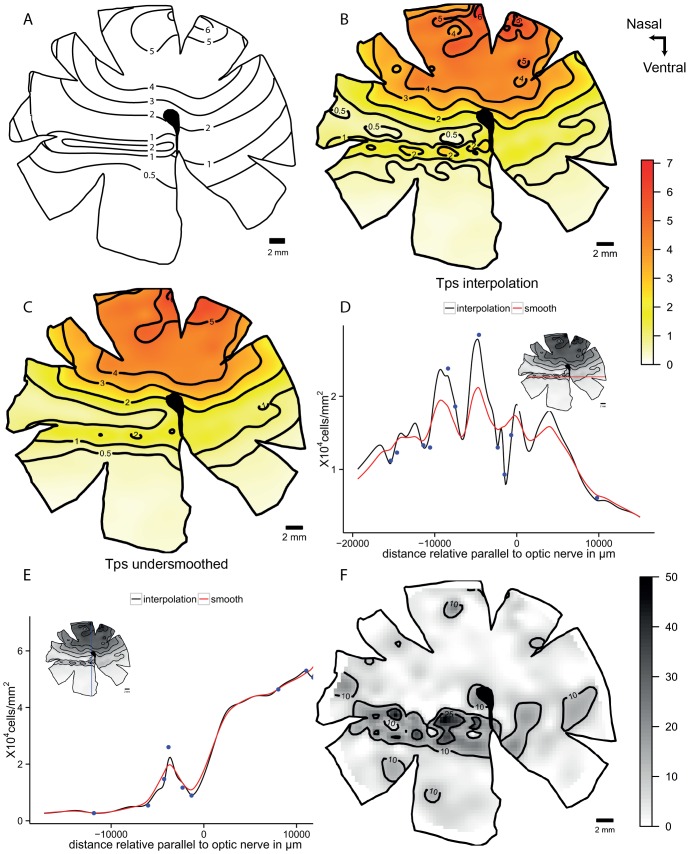
Topographic maps of the ganglion cells showing dorsal *area* specialization in *Gymnocranius bitorquatus*. Topographic maps of the ganglion cells in the retina of the collared sea bream, *Gymnocranius bitorquatus*, that show a dorsal area specialization. A. Modified map from Collin and Pettigrew, 1988b. B. Thin plate spline interpolation using a lambda value of 0. C. Thin plate spline under-smoothed map. D. Horizontal transect crossing the supposed streak specialization. E. Vertical transect crossing the highest cell density area, the dots represent observed points that are in the transect or at a distance not higher than 0.5 mm of the transect line. F. Residual map for the over-smoothed model showing percentage of variation between the calculated data and the observed data, the contour lines represent the areas that varied more than 10% and 25%. All the densities are ×10^4^ cells mm^−2^.

The collared sea bream, *Gymnocranius bitorquatus* shows a dorsal specialization with a central *area* and a lower density horizontal streak of 20,000 cells mm^−2^ in the centre of the retina. The interpolation model shows some isolated points but the streak lies within the 10,000 cells mm^−2^ contour that is clearly represented in the smoothed model with less local variation. The horizontal transect shows this variation in the streak with the oscillations between 10,000 and 20,000 cells mm^−2^. Note how the oscillations in the lines are reduced with the smoothed model indicating that the noise is reduced. The residual map shows that the highest level of variation is in the position of the horizontal streak. High fluctuations of the density values in the streak mean that the smoothing model will reduce these values (Figure 7).

**Figure 8:**
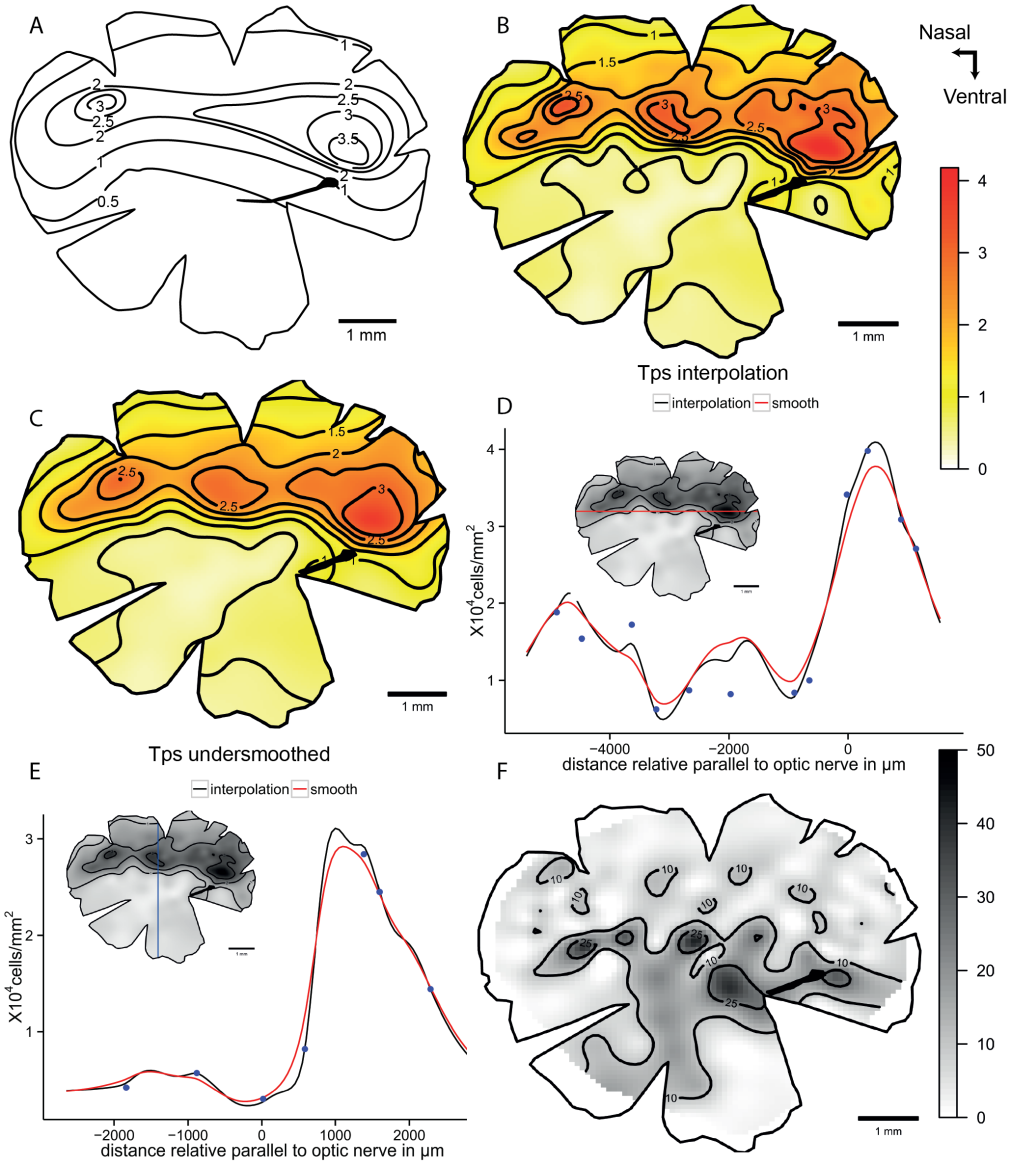
Topographic maps of the ganglion cells showing three areae specializations in *Parapercis cylindrica*. Topographic maps of the Ganglion cells in the retina of the sandperch, *Parapercis cylindrica*, that shows a horizontal streak with three *areae* specializations. A. Modified map from Collin and Pettigrew, 1988a. B. Thin plate spline interpolation using a lambda value of 0. C. Thin plate spline under-smoothed. D. Horizontal transect crossing the horizontal streak specialization. E. Vertical transect crossing the middle *area* specialization, the dots represent observed points that are in the transect or at a distance not higher than 0.5 mm of the transect line. F. Residual map for the over-smoothed model showing percentage of variation between the calculated data and the observed data, the contour lines represent the areas that varied more than 10% and 25%. All the densities are ×10^4^ cells mm^−2^.

The original topographic map of the sandperch, *Parapercis cylindrica*, possesses a horizontal visual streak with two areas of elevated cell density, one that is located in the temporal retina and one that is located in the nasal retina. The Tps model shows the same overall pattern as the original map, but the interpolation method reveals a third peak in the centre of the streak. The smoothed model shows a similar distribution in the topographic map and it produces more radial iso-density contours for a more aesthetically pleasing result. On the other hand, we can observe from the line transects that the higher values within the specializations are lowered by this model. Specifically, the nasal and temporal peaks have a lower magnitude, while the central peak is slightly higher. The vertical transect shows a nice comparison between the two models. Most of the variation in the points can be observed in the central area as well as some subregions within the streak (Figure 8).

### Special case - Fovea

**Figure 9:**
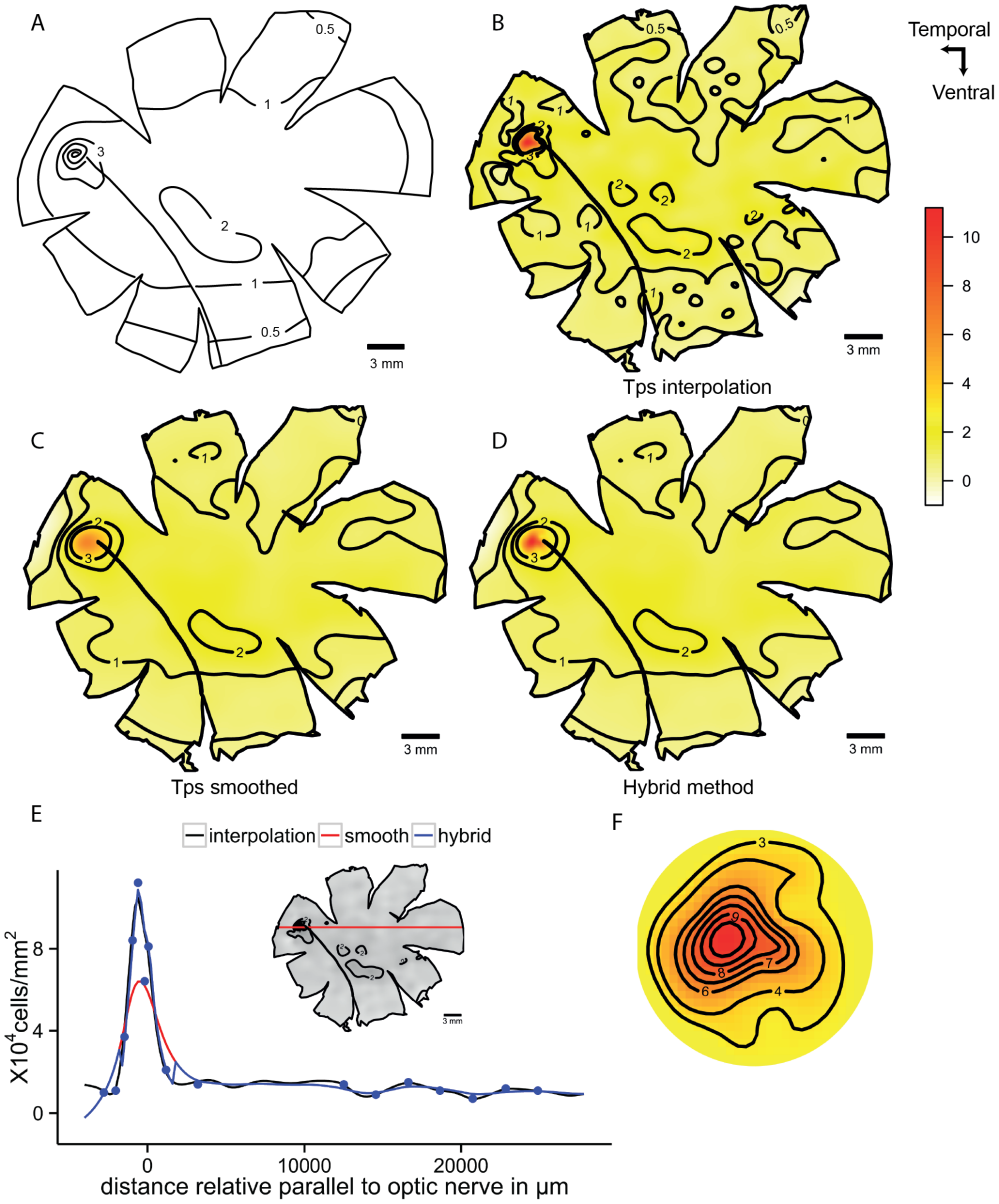
Topographic maps of the ganglion cells showing a *fovea* in *Conocara murrayi*. Topographic maps of the ganglion cells in the retina of the smooth-head fish, *Conocara murrayi*, which shows a *fovea*. A. Modified map from Collin and Patridge, 1996. B. Thin plate spline interpolation using a lambda value of 0. C. Thin plate spline under-smoothed. D. Hybrid model with Tps smoothed to GCV in the periphery and cubic interpolation in the fovea region. E. Horizontal transect crossing the *fovea*, the dots represent observed data 0.5 mm the transect line. F. Detail of the *fovea* region using Tps interpolation. All the densities are ×10^3^ cells mm^−2^.

The deep-sea smooth-head fish, *Conocara murrayi*, possesses a temporal *area* with a fovea. It has approximately ten times higher densities of cells than are found in the retinal periphery. The graphical representation and the spatial analysis of this type of retina can be challenging. When the data is smoothed using the algorithms described above, the high density points are reduced by close to half of the observed values. On the other hand, if an interpolation algorithm alone is used the maps display local fluctuations in the lower density areas that probably represent ‘noise’ in the count data. The hybrid method of smoothing the data in the periphery but interpolating the data in the fovea optimizes the assessment of cell density in these two different regions independently in order to accurately characterise a major specialization (fovea) while still reducing the noise in the retinal periphery. The transect line that crosses the fovea shows how the hybrid model uses interpolation to preserve the high values present in the fovea but it uses the smoothed algorithm to reduce low density fluctuations from the periphery. The difference between the values obtained by the smoothing and the interpolation methods is evident in the horizontal transect (Figure 9).

## Discussion

We have confirmed that the use of different models does not change the representation of the main retinal specializations, where the option of interpolate the data show an accurate map without modifying any value and the use of a smoother method (it is Gaussian kernel smoother or thin plate spline) will give a similar representation but reduces the noise and effects of potential outliers. The variation in the application of the smoothing parameters does affect the accuracy of the data presented and potentially its interpretation. The great diversity of retinal specializations constitutes a challenge to adopt a single model to compare the retinas but is highly important the use of an objective spatial analysis. The flexibility in the use of R allows the user to conduct the spatial analysis of retinal neuron distribution in different ways. This applies equally to the traditional method where retinal loci are examined using a compound microscope and manual counts of cell density are drawn onto a map, calibrated and iso-density contours constructed manually using limited linear interpolation or the more automated methods such as the software StereoInvestigator. This R script also gives the flexibility to change between different models of spatial analysis and to modify the smoothing parameters in order to optimize the accuracy of the final map. It also provides a faster, more reliable way to calculate and represent the topographic distribution of retinal neurons.

There are more than 30 different ways to analyse this type of data including the use of interpolations, kernel smoothers and spline methods to represent spatial distributions while taking into account things like clustering, independence of the areas, and nearest neighbour distances [46]. However, we found that for retinal analyses, where the sampling is relatively homogeneous and the distance between the observations is independent to cell density and the use of different smoothers does not alter the results. The smoothing models presented here help not only the interpretation of the data but lends visual support to which model to use in addition to improving the aesthetic appeal of the maps. The models analyse the relationship between the different observation points assessing the spatial position and magnitude of each observation to detect a spatial pattern and reduce possible outliers [60]. Smoothing models also help to reduce artefactual variation caused by retinal dissection and whole mounting [17]. The cuts used to flatten the retina, physically modify the cell densities close to the retinal edges. Removal of the retinal pigmented epithelium, the choroid and/or the vitreous humour might also stretch or damage the tissue and therefore alter local cell density. The use of a smoothing algorithm is a very effective way of reducing the ‘noise’ caused by such artefactual variations by analysing the spatial distribution of the observations. If the author does not know the source of natural or artefactual variation, it is always recommended to run the models with the default parameters [49], [56] and then compare those using residuals analysis or transects.

This is the first study that compares the use of different spatial analysis algorithms to assess the topographic distribution of neurons in the retina. It is important to consider the characteristics of the retina when choosing the right smoothing parameters because of the possibility of under- or over- smoothing, where the densities can change and consequently the representation and characterisation of the specialization. Each retina should be treated independently according to their morphological characteristics. For example, a retina that might be damaged during dissection and handling will produce high artefactual variation and should be slightly over-smoothed, thereby reducing the noise and showing the natural topographic variation. This, however, always comes at the cost of under-representing the peak density. In some cases, one might expect high natural variation and the minor fluctuations in the topographic distribution could be important. In this case, under-smoothing the retina would be a better approach as performed on the retinal data for the blue tusk fish, *Choerodon albigena*, where the under-smoothed model shows clearly the dorso-temporal specialization is evident and the horizontal streak is clearly present [4]. The type of the study might also require a different level of smoothness; a comparative study between different species might not be affected by over-smoothing the retina while a study to identify small variability within organisms of the same species might use under-smoothing parameters.

The numerical output of each model for subsequent assessment of the maps depends on the type of algorithm used to analyse the data. Gaussian kernel smoothing using the *spatstat* package analyses only the points inside the retina, i.e. it creates a window, where the points are analysed and the cuts used to flatten the retina are considered to be empty spaces. It is possible to make very complex windows with holes and multiple polygons, which is particularly useful for the spatial analysis of the retina. The function in *spatstat* also has an edge corrector to fill the gaps between the last observed points in the periphery and the edge of the retina. It also gives a nice graphical representation because the final output is an image object instead of a numerical function, but this reduces the ability to perform subsequent statistical analyses [56], [58]. However, the values calculated by the model can be extracted from the image using a raster model.

Thin plate spline, is a very powerful model that provides a *kriging* object as an output, which can predict the value at any coordinate. We prefer this model because it allows one using linear interpolation with a lambda value of 0 to assess different levels of smoothness. The use of degrees of freedom to modify the smoothness is more interpretative than the use of different lambda values. One of the disadvantages of this model is that it has to be masked to obtain the contour of the retina and can be computationally-intensive and thus slow to run when constructing high resolution maps with thousands of points because each point is a pixel that represents a value. The Tps model has the advantage that the data frames obtained can be used easily for both statistical and graphical analyses [49]. The study of the retinal specializations requires the analysis of the highest densities areas [4], [8]. Most of the smoothing models tend to pull the highest and lowest values towards the mean of the population. Nevertheless, the models tend to provide complex spatial analysis, where local variation is analysed and higher values are respected. However, the use of a lower level of smoothness will give a more realistic value for the streak while, at the same time, maintaining natural local variability.

Comparing traditional methods of creating topographic maps, where the limitation of calculating the interpolation between some specific points over a spline interpolation using all the sampled points, can give slightly different results with more resolution obtained with the spline interpolation. In *P. cylindrica*, only two zones are described in the original paper [20] but using the thin plate spline model described here, it clearly shows a horizontal streak with three zones. Similarly, *G. bitorquatus* there is a well defined dorsal *area* with similar values obtained in the original map and the smoothed model. However, the original map shows a horizontal streak of higher cell density than the thin plate spline model. This is due to the streak not being continuous so the points cannot be connected in a streak. On the contrary, the smoothing model places more emphasis on the spatial arrangement of the cells, which consequently means that the horizontal streak has a lower cell density [4].

The unbiased construction of iso-density contour lines, which may be effective in helping to characterise a retinal specialisation, can be obtained by the thin plate spline models. Without the most accurate data (especially within the regions of highest cell density) and without the use of colour gradients, it may be difficult to assess the spatial arrangement and relative importance of a retinal acute zone. In *C. miniatus*, the nasal and temporal specialisations have marked differences in cell density. In the original map, the iso-density contour lines for the nasal specialisation include a 15,000 cells mm^−2^ and a 20,000 cells mm^−2^ contour in contrast to the temporal specialisation, which shares these same contours but peaks with a density of 40,000 cells mm^−2^. This may confound the importance of the two acute zones. The text of the original paper recalls this difference but readers looking for quick reference might look only for the graphical representation [20].

The fovea represents a special case because it has a high natural gradient in addition to the presence of a localised invagination, where the interpolation model gives a lot of noise in the periphery [5]. The subsampling of the foveal region and the analysis using cubic interpolation respects the high values of the fovea and reveals the real density values for the specialization. It is, therefore, possible to represent the fovea independently and plot the iso-density contour lines separately and combine the data by removing the points from the smoothed model in the area of the fovea and merging the interpolated predictions (Figure 9D).

Previous studies have analysed topographic densities using statistical software that might be expensive or inaccessible. The current script runs in a freely available open source program (R) and can be edited as required for specific purposes. The use of a continuous colour gradient is innovative and improves the interpretation of the variation inside the contour intervals. The current study uses a ‘heat gradient’ representation that goes from white at low densities to red at high densities. This is useful because heat colours when transformed to black and white still present the same light to dark gradient. Additionally there are two colour gradients in the script that can be used in different circumstances: 1) The rainbow gradient from the *timcolours* package, where blue represents low values and red represents high values, which shows a nice representation of the densities that might be useful for visual presentations and 2) The grey gradient that might be used to for black and white publications [49], [61]. The use of the R script has additional advantages, such as the easy manipulation of the maps. The commands are quick and any parameter can be changed easily with no need to repeat the whole process. Some parameters can be modified for better representation of the data including the number of contour lines and the intervals between the contours, the colour gradients of the map, the size of the text and lines and the x, y and z coordinate limits that are useful for creating the correct representation of the maps. The process to standardize the topographic maps to make them comparable [62] could be greatly improved by the use of the current script. If future studies give information in the observations and the position in the maps, more statistical analysis can be applied to extract more information on a global scale.

We consider that the main benefits of this method are in identifying specialisations that might be missed using conventional techniques and/or in reducing noise in the map patterns that are the result of random fluctuations or artefacts. This method also formalizes the analysis such that statistical comparisons can be made to robustly represent topographic data, which is both accurate and reproducible. Each retinal map should be analysed by a formal spatial analysis but it should be tested against transects or residuals to show that the smoothing parameter is accurate to the observed data.

## Supporting Information

File S1 **retina.xml.** File of the Pacific spookfish, *Rhinochimaera pacifica* obtained by exporting the StereoInvestigator information to a xml format file.
(XML)

File S2 **One cell map V8.R.** File of the script to analyse retinas with the xml format using R software program. The file should be copied in the same directory than the xml file.
(R) Click here for additional data file.

File S3 **A curacao 23.svg.** File of the staghorn damselfish, *Amblyglyphidodon curacao* obtained by digitalizing the original data published in Collin and Pettigrew, 1988.
(SVG)

File S4 **One cell map V8 svg version.R.** File of the script to analyse retinas with thesvg format using R software program. The file should be copied in the same directory than the svg file.
(R) Click here for additional data file.

File S5 **Collin and Pettigrew 1988.pdf.** Paper where the original maps of the coral cod, *Cephalopholis miniatus*; the staghorn damselfish, *Amblyglyphidodon curacao*; and the sandperch, *Parapercis cylindrica*. Collin SP, Pettigrew JD (1988) Retinal Topography in Reef Teleosts I. Some Species with Well-Developed Areae but Poorly-Developed Streaks. Brain, Behavior and Evolution 31: 269–282.
(PDF)

File S6 **Collin and Pettigrew 1988b.pdf.** Paper where the original maps of the blue tusk fish, *Choerodon albigena*; and the collared sea bream, *Gymnocranius bitorquatus*. Collin SP, Pettigrew JD (1988) Retinal Topography in Reef Teleosts II. Some Species with Prominent Horizontal Streaks and High-Density Areae. Brain, Behavior and Evolution 31: 283–295.
(PDF)

File S7 **Collin and Partridge 1996.pdf.** Paper where the original map of the smooth-head fish, *Conocara murrayi*. Collin SP, Partridge JC (1996) Retinal specializations in the eyes of deep-sea teleosts. Journal of Fish Biology 49: 157–174.
(PDF)
