# Supplementary material for: A Comparison of Spatial Analysis Methods for the Construction of Topographic Maps of Retinal Cell Density
Source: PLoS One. 2014 Apr 18;9(4):e93485. doi: 10.1371/journal.pone.0093485 (PMC3998654; doi:10.1371/journal.pone.0093485)
Supplement: File S5 — Collin and Pettigrew 1988.pdf. Paper where the original maps of the coral cod, Cephalopholis miniatus; the staghorn damselfish, Amblyglyphidodon curacao; and the sandperch, Parapercis cylindrica. Collin SP, Pettigrew JD (1988) Retinal Topography in Reef Teleosts I. Some Species with Well-Developed Areae but Poorly-Developed Streaks. Brain, Behavior and Evolution 31: 269–282. (PDF) [file pone.0093485.s005.pdf]

Brain, Behavior and Evolution
Editor-in-Chief: R. Glenn Northcutt, La Jolla, Calif.

Reprint
Publisher: S. Karger AG, Basel
Printed in Switzerland

Brain Behav. Evol. *31:* 269–282 (1988)

Retinal Topography in Reef Teleosts

I. Some Species with Well-Developed Areae but Poorly-Developed Streaks

Shaun P. Collin, John D. Pettigrew

Neuroscience Laboratory, Department of Physiology and Pharmacology, University of Queensland, St. Lucia, Qld., and Heron Island Research Station, University of Queensland, Heron Island via Gladstone, Qld., Australia

Key Words. Fish · Retina · Ganglion cell · Area centralis · Vision

Abstract. The retinal ganglion cell layer of five species of teleosts has been studied from Nissl-stained wholemounts and the distribution of neuronal elements determined quantitatively. Isodensity contour maps of neurons in the ganglion cell layer revealed areas of high density (areae centrales) predominantly in the temporal retina, but other areae were also found in the nasal and dorso-nasal retina. Neuronal densities within the ganglion cell layer at the areae centrales ranged from 0.4×10^4 to 4.7×10^4 cells/mm^2. Species that were found to lack a horizontal streak of high ganglion cell density appear to be those whose behaviour suggests they possess an interrupted view of the sand-water horizon and are 'enclosed' species. Concentric density contours around an area centralis seem to be associated with enclosed environments. The relationship between retinal topography and niche is also discussed.

Introduction

Topographic analysis of the retinal ganglion cell layer has yielded valuable information on the visual capabilities of a variety of vertebrates and invertebrates. The wholemount technique [Stone, 1965] enables neuronal elements within the ganglion cell layer to be stained and visualised. Areas of increased density have often been the subject of investigation in mammals [Hughes, 1977], and these areas have been thought to be retinal regions having greater resolving power than the remainder of the retina [Rolls and Cowey, 1970; Rodieck, 1973; Frisen and Frisen, 1976].

Gulliver [1868] was the first to report a 'fovea' in fish and later described a well developed retinal pit in the sea horse, *Hippocampus*. Slonaker [1897] and Kahmann [1934, 1935] presented a comparison of vertebrate classes possessing an area of acute vision. These early works were qualitative, and only recently has the resolution of acute zones been subject to quantitative analysis. Such analysis of the retinal ganglion cell layer in teleosts has so far been restricted to four species: the coral fish, *Microcanthus strigatus* [Yamanouchi, 1956], the kelp bass, *Paralabrax clathratus* [Schwassmann, 1968], the scorpaenid, *Sebastiscus marmoratus* [Ito and Murakami, 1984] and a filefish, *Navodon modestus* [Ito and Murakami, 1984].

In this study we have extended such analyses to the retinal topography of five species from five representative families of reef teleosts, occupying a selected range of niches. The species that we present in this study have either absent or poorly-developed horizontal retinal 'streaks'. In a companion paper [Collin and Pettigrew, 1988] we provide similar data for five different teleost species, each of which shows greater development of the horizontal streak. In addition to forming a basis for subsequent investigations of visual pathways and brain organisation, these analyses extend our knowledge of vertebrate retinal topography [Hughes, 1971, 1974; Hebel, 1976; Fukuda, 1977; Provis, 1979; Tancred, 1981]. They also provide some new bases for testing ideas about the ecological and functional roles of topographic specialisations, such as the 'terrain theory', which suggests that the distribution of ganglion cells across the retina depends on the symmetry of the perceived world [Hughes, 1977].

Table I. Summary of the habitat and methods of feeding of the five species under investigation, compiled from Carcasson [1977], Grant [1982] and direct observations

Species	Family	Preferred habitat	Method of feeding	Position of area	Cells × 10^4/mm^2
Halophryne diemensis (Australian frogfish)	Batrachoididae	caves or crevices	rapid flexion of oblique mouth, camouflage provides surprise	temporal<br>weak horizontal streak	0.7<br>0.4
Pomacanthus semicirculatus (blue angel fish)	Pomacanthidae	caves, under coral overhangs	chisel-shaped teeth scrape away encrusting sessile organisms	temporal<br>nasal	1.4<br>1.3
Amblyglyphidodon curacao (staghorn damsel fish)	Pomacentridae	amongst branches of staghorn coral	diurnal omnivore of zooplankton	temporal<br>ventro-temporal<br>dorso-nasal	2.5<br>3.0<br>2.3
Parapercis cylindrica (sharp-nosed weever fish)	Parapercidae	on sand and in areas of coral rubble	voracious carnivore, seeking prey while perched on large pectoral fins	dorso-temporal<br>dorso-nasal	4.0<br>3.4
Cephalopholis miniatus (coral cod)	Serranidae	caves and under coral overhangs	ambush feeder from within dark caves	temporal<br>nasal	4.7<br>2.2

The retinal positions of areas of highest cell densities (areae centrales) are given with their corresponding cell densities.

Materials and Methods

A summary of the five teleost species investigated is presented in table I. Brief descriptions of species' niche and feeding strategies are provided for comparison. Three adults were used in each case, allowing six retinal examinations per species.

Technique

After immersion in MS 222 (100 mg/liter salt water), each fish was sacrificed by an intramuscular injection of Rompun (xylazine, 0.025 ml). Transcardial perfusion with saline followed, and both eyes were then removed, fixed in 4% formaldehyde in 0.1 *M* phosphate buffer and stored. After 4 weeks, the cornea, lens and vitreous were removed, and small incisions were made on the perimeter of the retina for temporal, nasal, dorsal and ventral orientation. The falciform process, an elongated extension of the choroid, was severed from the sclera and the retina removed. A pair of No. 5 watchmaker's forceps were used to dissect away the pigment layer, and a number of radial incisions were made to allow the retina, ganglion cell layer uppermost, to be mounted on a gelatinised slide. After the wholemount was dried for 12 h, the ganglion cell layer was stained for Nissl substance in cold 0.05% cresyl violet (pH = 4.3, acetate buffer) for 8–12 min to allow penetration of the thick optic nerve fibre layer found in many teleost retinae.

Fig. 1. The upper diagram in this and figure 2, 3, 4 and 7 illustrates the iso-density contour map of cells within the ganglion cell layer over the left retina, and in this case of *Pomacanthus semicirculatus* (Pomacanthidae, Actinopterygii). A temporal zone of 1.4×10^4 cells/mm^2 and a nasal zone of 1.3×10^4 cells/mm^2 are aligned along an axis, tilted dorsally 30° from the horizontal. These retinal specialisations reveal low density gradients and low visual acuity. The lower left diagram illustrates the lateral compression of this carnivore which possesses highly mobile eyes and relies on its rasping teeth to scrape encrusting organisms from rocks, from within caves and underneath coral overhangs. Scale bar = 50 mm. In this and figure 2, 3, 4 and 7, the lower right figure is a schematic representation of the marine habitat on the reef, viewing a cross-section of the various niches inhabited by the range of teleosts in this study. *P. semicirculatus* is depicted as a silouette to illustrate its preference for densely vegetated coral zones or under coral overhangs. Areas to the left of this figure depict caves and enclosed areas close to the reef crest and areas closer to the surface with a preference for staghorn coral. The right side depicts the more open areas of sand and coral, interposed with isolated coral outcrops, which would provide some protection for an organism escaping predation.

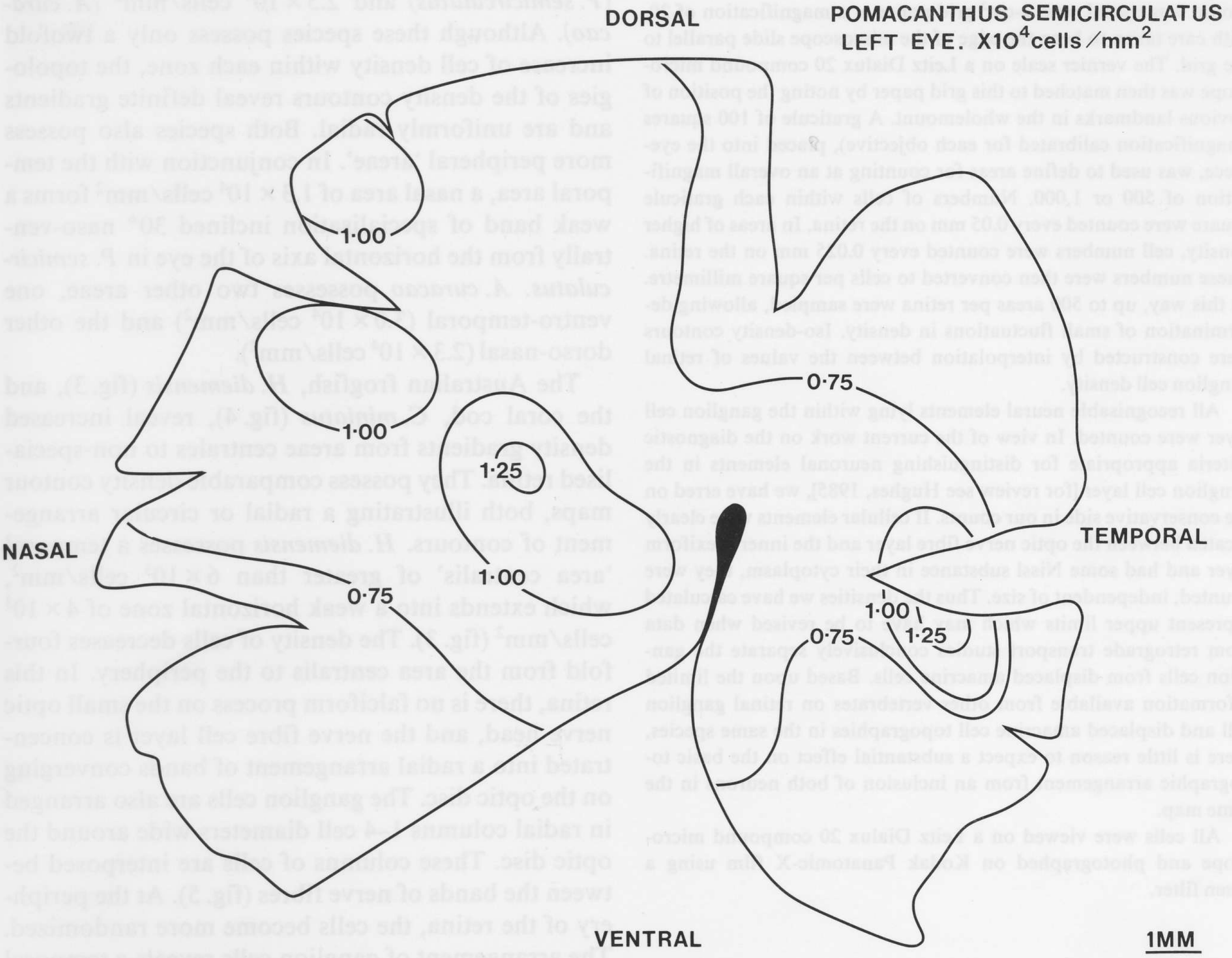
DORSAL
POMACANTHUS SEMICIRCULATUS
LEFT EYE. X10⁴ cells/mm²
NASAL
TEMPORAL
VENTRAL
1·00
1·00
1·25
1·00
0·75
0·75
1·00
0·75
1·25
1MM

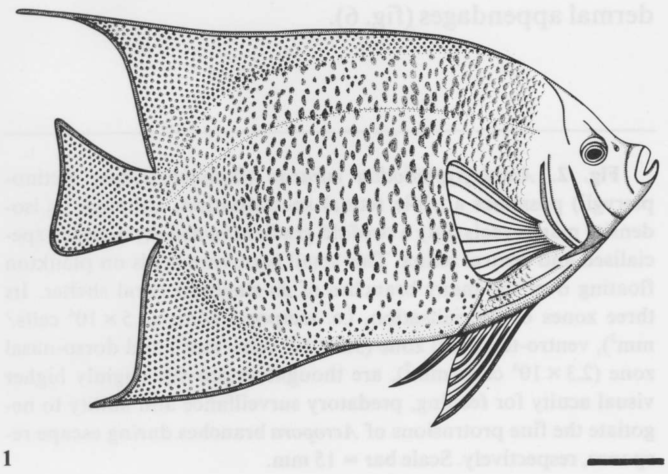

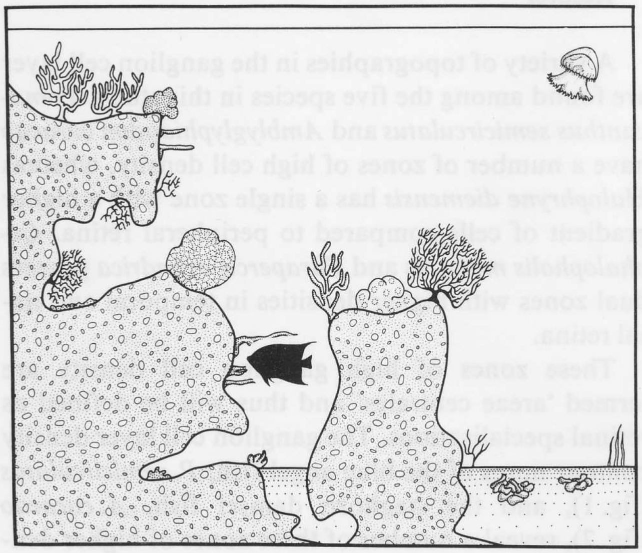

1

Densities of stained cells were determined for both eyes, although only the left eye preparations are presented here. Using a Jena DK2 microfiche reader, the outline of each retinal wholemount was traced onto 1-cm^2 grid paper at a magnification of 20, with care taken to keep the edge of the microscope slide parallel to the grid. The vernier scale on a Leitz Dialux 20 compound microscope was then matched to this grid paper by noting the position of obvious landmarks in the wholemount. A graticule of 100 squares (magnification calibrated for each objective), placed into the eyepiece, was used to define areas for counting at an overall magnification of 500 or 1,000. Numbers of cells within each graticule square were counted every 0.05 mm on the retina. In areas of higher density, cell numbers were counted every 0.025 mm on the retina. These numbers were then converted to cells per square millimetre. In this way, up to 500 areas per retina were sampled, allowing determination of small fluctuations in density. Iso-density contours were constructed by interpolation between the values of retinal ganglion cell density.

All recognisable neural elements lying within the ganglion cell layer were counted. In view of the current work on the diagnostic criteria appropriate for distinguishing neuronal elements in the ganglion cell layer [for review see Hughes, 1985], we have erred on the conservative side in our counts. If cellular elements were clearly located between the optic nerve fibre layer and the inner plexiform layer and had some Nissl substance in their cytoplasm, they were counted, independent of size. Thus the densities we have calculated represent upper limits which may have to be revised when data from retrograde transport studies conclusively separate the ganglion cells from displaced amacrine cells. Based upon the limited information available from other vertebrates on retinal ganglion cell and displaced amacrine cell topographies in the same species, there is little reason to expect a substantial effect on the basic topographic arrangement from an inclusion of both neurons in the same map.

All cells were viewed on a Leitz Dialux 20 compound microscope and photographed on Kodak Panatomic-X film using a green filter.

Results

A variety of topographies in the ganglion cell layer are found among the five species in this study. *Pomacanthus semicirculatus* and *Amblyglyphidodon curacao* have a number of zones of high cell density, whereas *Halophryne diemensis* has a single zone with a higher gradient of cells compared to peripheral retina. *Cephalopholis miniatus* and *Parapercis cylindrica* possess dual zones with higher densities in temporal and nasal retina.

These zones of high ganglion cell density are termed 'areae centrales' and thus will be defined as retinal specialisations. The ganglion cell layer density contour maps of the blue angel fish, *P. semicirculatus* (fig. 1), and the staghorn damsel fish, *A. curacao* (fig. 2), reveal a number of these zones of higher density. The areae centrales situated in temporal retinae possess cell densities in excess of 1.4×10^4 cells/mm^2 *(P. semicirculatus)* and 2.5×10^4 cells/mm^2 *(A. curacao)*. Although these species possess only a twofold increase of cell density within each zone, the topologies of the density contours reveal definite gradients and are uniformly radial. Both species also possess more peripheral 'areae'. In conjunction with the temporal area, a nasal area of 1.3×10^4 cells/mm^2 forms a weak band of specialisation inclined 30° naso-ventrally from the horizontal axis of the eye in *P. semicirculatus*. *A. curacao* possesses two other areae, one ventro-temporal (3.0×10^4 cells/mm^2) and the other dorso-nasal (2.3×10^4 cells/mm^2).

The Australian frogfish, *H. diemensis* (fig. 3), and the coral cod, *C. miniatus* (fig. 4), reveal increased density gradients from areae centrales to non-specialised retina. They possess comparable density contour maps, both illustrating a radial or circular arrangement of contours. *H. diemensis* possesses a temporal 'area centralis' of greater than 6×10^3 cells/mm^2, which extends into a weak horizontal zone of 4×10^3 cells/mm^2 (fig. 3). The density of cells decreases fourfold from the area centralis to the periphery. In this retina, there is no falciform process on the small optic nerve head, and the nerve fibre cell layer is concentrated into a radial arrangement of bands converging on the optic disc. The ganglion cells are also arranged in radial columns 1–4 cell diameters wide around the optic disc. These columns of cells are interposed between the bands of nerve fibres (fig. 5). At the periphery of the retina, the cells become more randomized. The arrangement of ganglion cells reveals a temporal area centralis and roughly concentric iso-density contours encompassing the entire retina. The eyes are large and frontal, and the head features three rows of dermal appendages (fig. 6).

Fig. 2. *Amblyglyphidodon curacao* (Pomacentridae, Actinopterygii) possesses a triple retinal specialisation, although its iso-density map reveals only a twofold gradient in density between specialised and central retina. This small carnivore feeds on plankton floating over the many branches of its staghorn coral shelter. Its three zones of specialisation, i.e. temporal zone, (2.5×10^4 cells/mm^2), ventro-temporal zone (3.0×10^4 cells/mm^2) and dorso-nasal zone (2.3×10^4 cells/mm^2), are thought to provide slightly higher visual acuity for feeding, predatory surveillance and ability to negotiate the fine protrusions of *Acropora* branches during escape response, respectively. Scale bar = 15 mm.

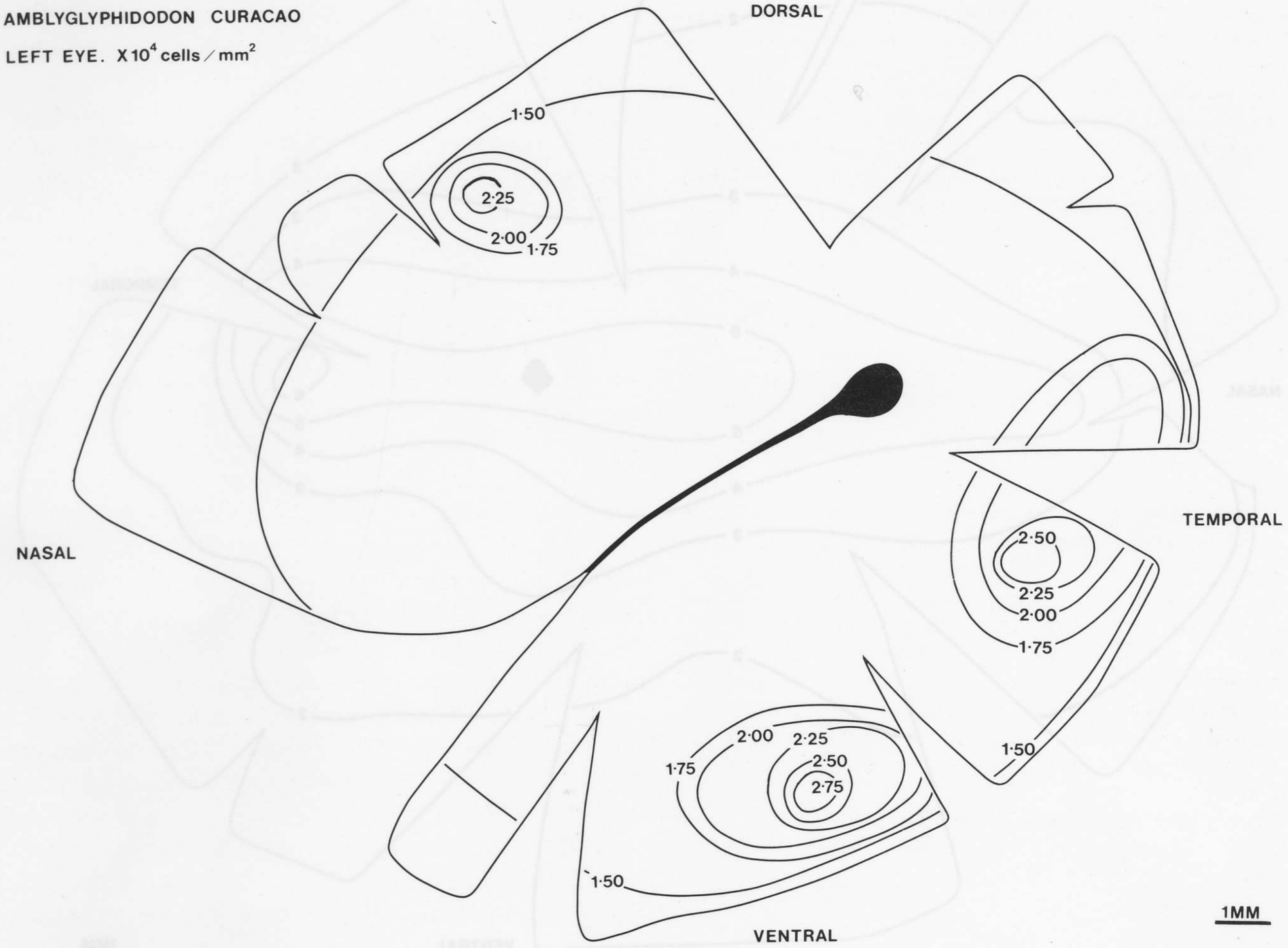
AMBLYGLYPHIDODON CURACAO
LEFT EYE. X10^4 cells / mm^2
DORSAL
NASAL
TEMPORAL
VENTRAL
1·50
2·25
2·00
1·75
2·50
2·25
2·00
1·75
1·50
2·00
2·25
2·50
2·75
1·75
1·50
1MM

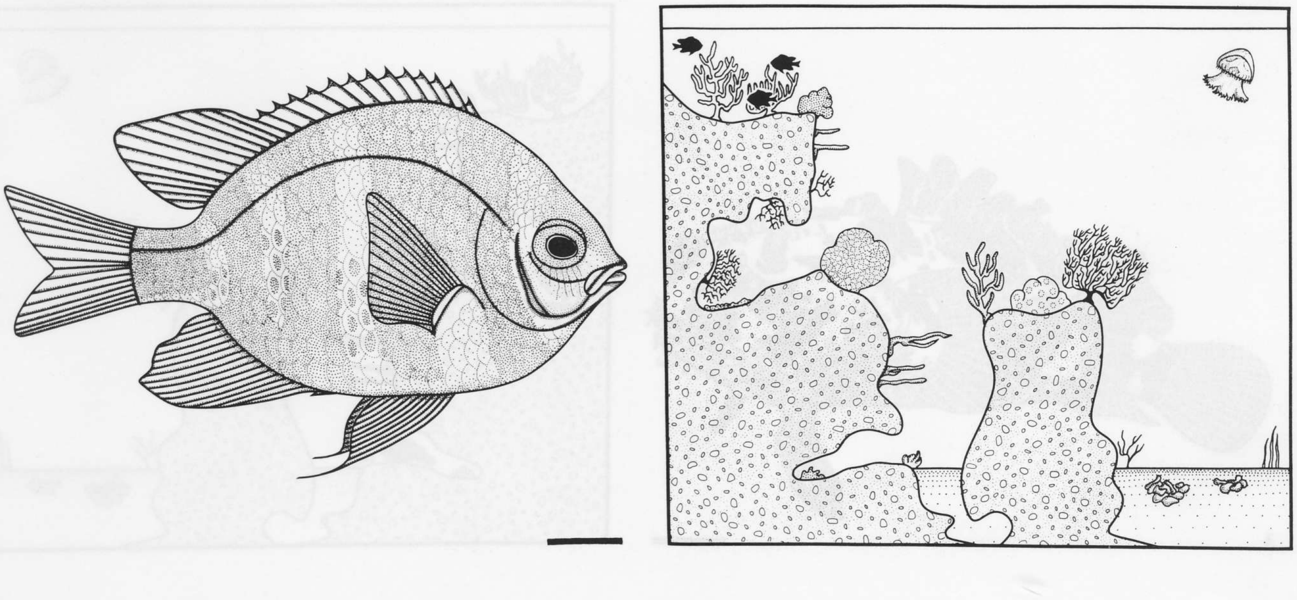

2

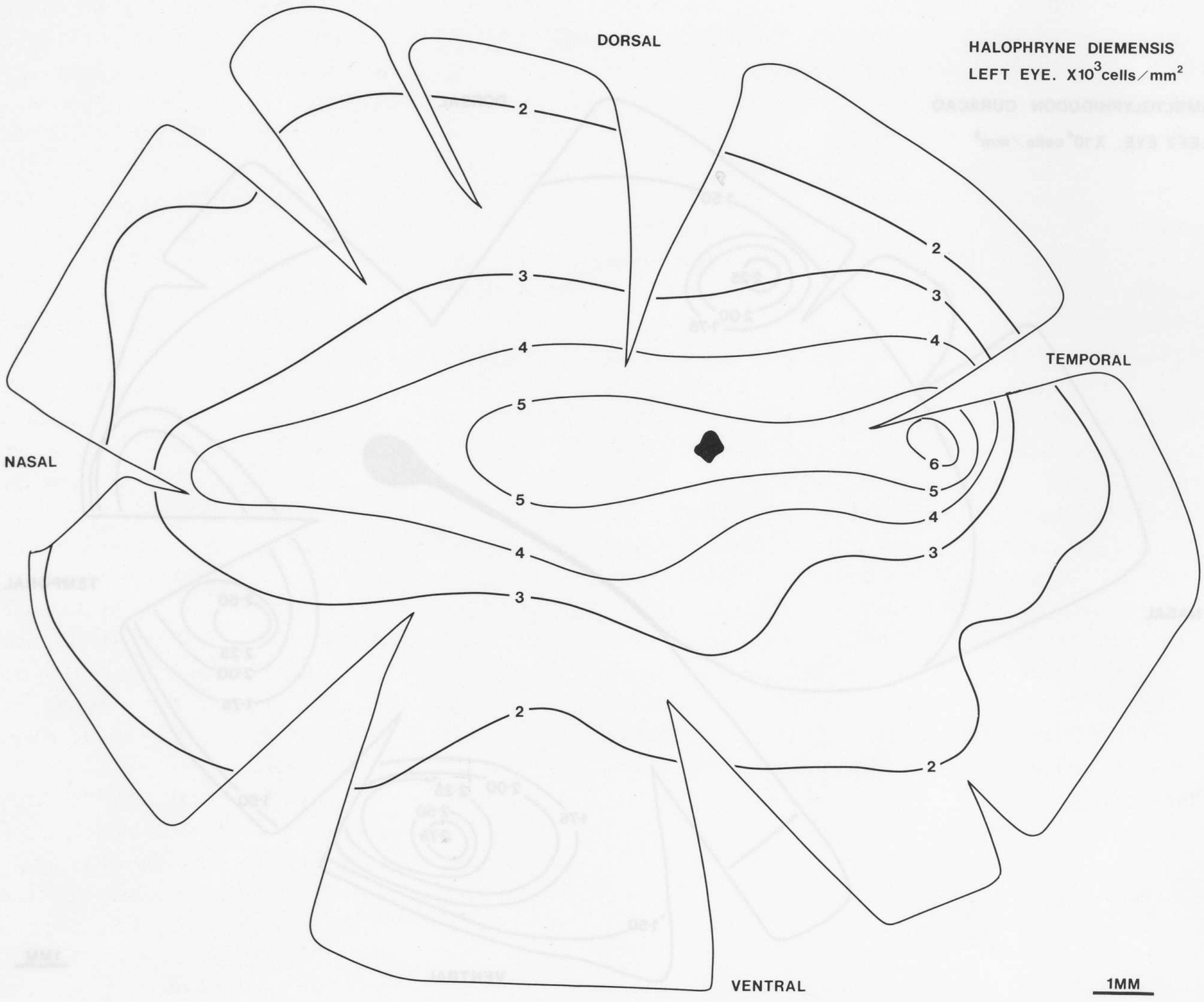
DORSAL
HALOPHRYNE DIEMENSIS
LEFT EYE. X10^{3}cells/mm^2
NASAL
TEMPORAL
VENTRAL
1MM
2
3
4
5
6

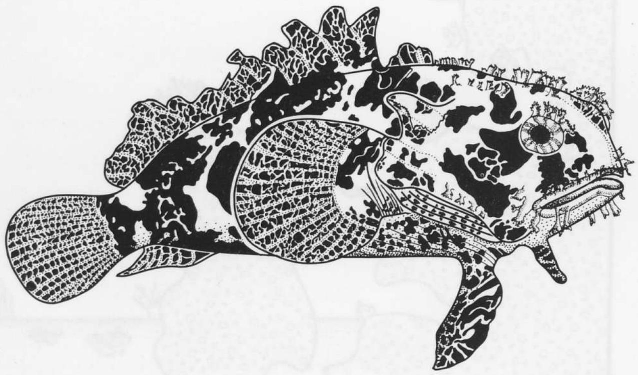

3

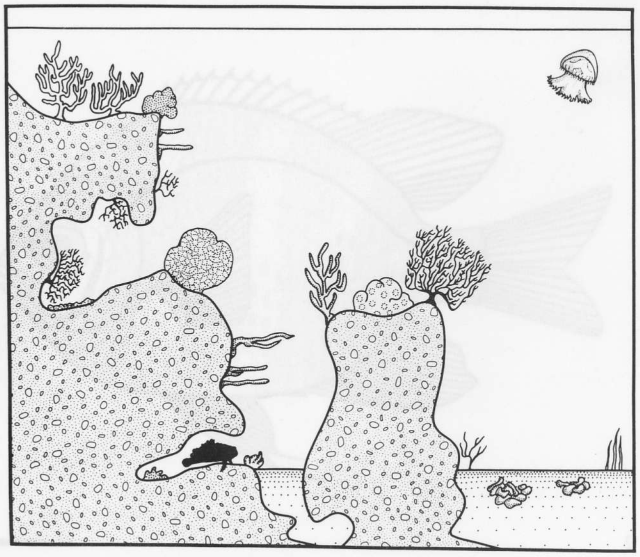

C. miniatus possesses a fourfold increase in cell density within an area centralis (4.7×10^4 cells/mm^2) close to the temporal margin of the retina (fig. 4). In *C. miniatus* and *H. diemensis*, the position of the temporal areae centrales relative to the position of the eyes presupposes a large binocular overlap. A second area, with a peak density of 2.2×10^4 cells/mm^2, exists on the nasal margin of the retina of *C. miniatus*. The acute zones of this dual specialisation are arranged on either end of the horizontal meridian and subtend an angle of 140°.

The weever fish, *P. cylindrica* (fig. 7), reveals an unusual retinal specialisation with two widely separated zones of increased ganglion cell density, a dorso-temporal area centralis (4.0×10^4 cells/mm^2) and a dorso-nasal area centralis (3.4×10^4 cells/mm^2), which subtend an angle of approximately 130°. This species, although found to possess dual specialisations both with circular arrangements of contours, also revealed a naso-temporal extension of contours or a weak horizontal streak just above the retinal meridian of the eye [Collin and Pettigrew, 1988].

The Nissl-stained ganglion cells of all species studied are of varying size and reveal many degrees of packing in areas of increased density (fig. 8). Soma areas and cell size classes will be the subject of a further study.

Discussion

Australian Frogfish

The Australian frogfish, *Halophryne diemensis*, is an extraordinary bottom dweller, a voracious carnivore with large oblique mouth and frontal eyes (fig. 6). It rests on foot-like pectoral fins while lying in wait for small fishes and crustaceans. As its name suggests, the frogfish has a number of amphibian-like features. In contrast to the myotome bundles of other teleosts, the musculature is separated into connective tissue membranes or aponeuroses, and the epidermis is loose-fitting and scaleless. The frogfish can also exist out of water for extended periods of time, emits loud croaking mating calls and gives birth to live young with a tadpole-like appearance [Carcasson, 1977]. Finally, we have found that it has an amphibian-like retina (fig. 3). This retina has a small circular optic nerve head, no falciform process, and a radial arrangement of fibres and cells.

Similar retinal columns and the absence of a falciform process have also been described in the retina of the clawed toad, *Xenopus laevis* [Graydon and Giorgi, 1984]. In both cases, the ganglion cells radiate toward the extremities of the retina, though the cells become more randomized at the periphery. Similar patterns of cell column distribution are found in other anurans *(Neobatrichus pelobatoides* and *Pseudophryne quentheri)* [L.D. Beazley, personal commun.], in rodents [Perry and Cowey, 1982] and in primates [Stone and Johnston, 1981], but not in any other advanced teleosts of which we are aware. Therefore, the absence of a falciform process, the existence of radial columns of ganglion cells within the retina, together with a number of phenotypic similarities to the anurans, raise some doubt as to the current placement of *H. diemensis* within the Actinopterygii.

The retino-tectal projections of this species (manuscript in preparation) and another closely related member of the Batrachoidiformes, *Opsanus tau* [Ebbesson, 1968] also reveal a more primitive configuration in tectal labelling than the group with which it is placed [Greenwood et al., 1966]. It is possible that this group may represent an earlier lineage from actinopterygian phylogeny.

Both *Halophryne* and *Xenopus* possess a weak horizontal streak across the horizontal meridian of the retina and, upon closer examination of the results of Graydon and Giorgi [1984], there is also a higher density of ganglion cells in temporal retina of *Xenopus*. The density of ganglion cells within this temporal specialisation is slightly higher in the frogfish (0.6×10^4 cells/mm^2, in comparison to 0.5×10^4 cells/mm^2 in *Xenopus*), while densities across the horizontal streak are comparable. However, whether this weak retinal specialisation is also functionally comparable, remains unknown. *Xenopus* relies minimally on vision for survival and possesses only $52–59 \times 10^4$ fibres within its optic nerve [Wilson, 1971]. *Halophryne* possesses 45×10^4 cells in the ganglion cell layer which is low in comparison to other teleosts, especially consid-

Fig. 3. The iso-density contour map of the Australian frogfish, *Halophryne diemensis* (Batrichoididae, Paracanthopterygii), reveals a temporal zone of 7×10^3 cells/mm^2 and a weak horizontal streak of 4×10^3 cells/mm^2 with radial isotropy. Note that the optic nerve is devoid of a falciform process (see fig. 5) in contrast to all other teleosts in this study. *H. diemensis* is depicted as a silhouette in a position illustrating its preference for caves or densely vegetated coral zones. Scale bar = 30 mm.

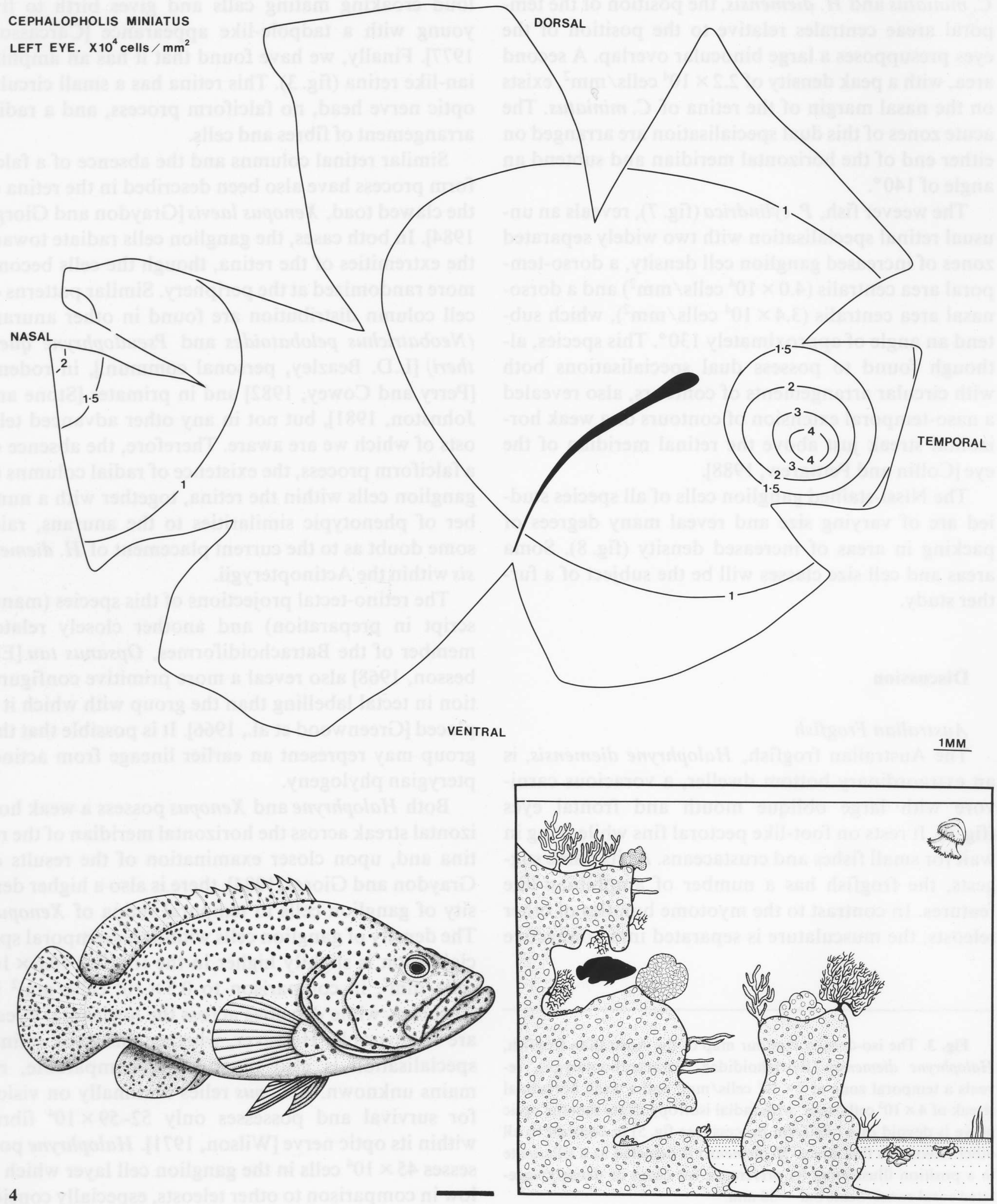
CEPHALOPHOLIS MINIATUS
LEFT EYE. X10³ cells / mm²
DORSAL
NASAL
TEMPORAL
VENTRAL
1MM
1
1·5
2
3
4

4

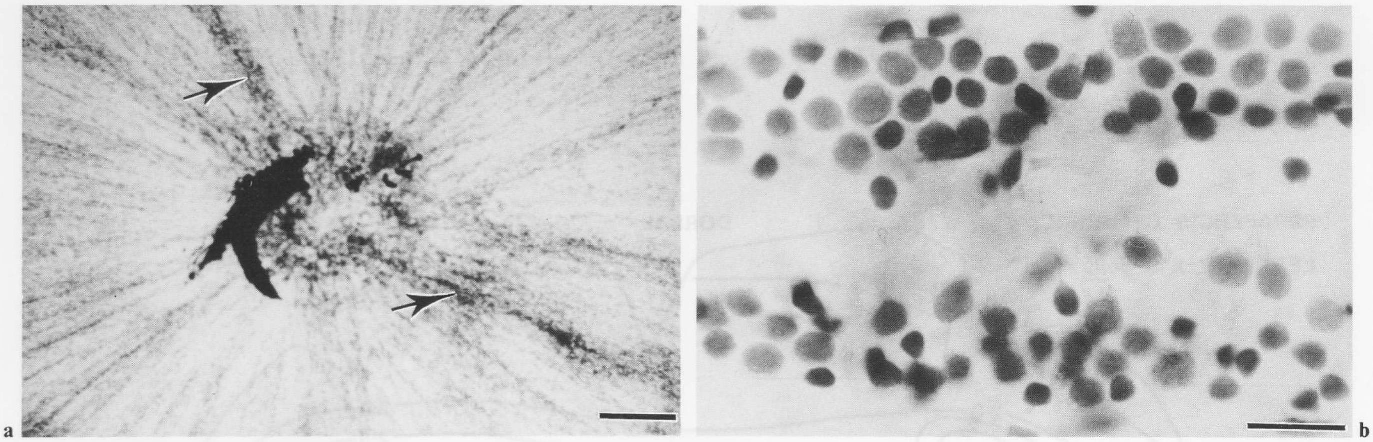

Fig. 5. **a** Anuran-like radial arrangement of cells around the optic nerve of the frogfish *Halophryne diemensis*. The optic nerve head contains a number of retinal blood vessels (arrowed) throughout the retina. Columns of Nissl-stained neurons in the ganglion cell layer are found to radiate towards the periphery. A fragment of the pigment epithelium remains adhered to the optic disc. Note the complete absence of a falciform process. Scale bar = 200 μm. **b** An enlargement of two radial columns of neurons in the ganglion cell layer from a region close to the optic nerve head. Cells become more random towards the periphery. Bundles of nerve fibres interpose between these columns of retinal neurons. Scale bar = 20 μm.

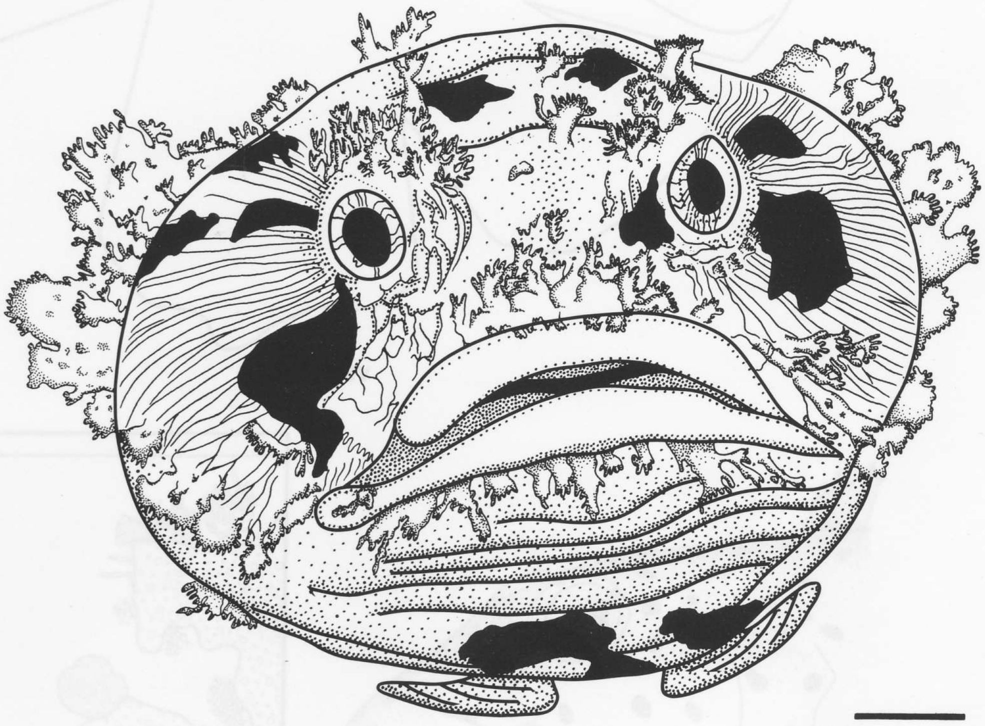

Fig. 6. Frontal view of the Australian frogfish, *Halophryne diemensis*. The arrangement of frontally positioned eyes and large oblique mouth closely resembles the appearance of some anurans. Three lateral lines extend over the head, each pore decorated with a cryptic dermal appendage. Scale bar = 10 mm.

Fig. 4. The coral cod, *Cephalopholis miniatus* (Serranidae, Actinopterygii), is an ambush hunter from within dark caves, preying on small fish which are engulfed by its prominent jaws. Its retinal iso-density map supports high eye manoeuvrability with two retinal specialisations, a temporal zone (4.7×10^4 cells/mm^2) and a nasal zone (2.2×10^4 cells/mm^2). The marked radial isotropy of its iso-density contours is thought to be a prerequisite of species that visualise a close three-dimensional environment where the horizon is not important or obscured by virtue of its chosen niche. Scale bar = 50 mm.

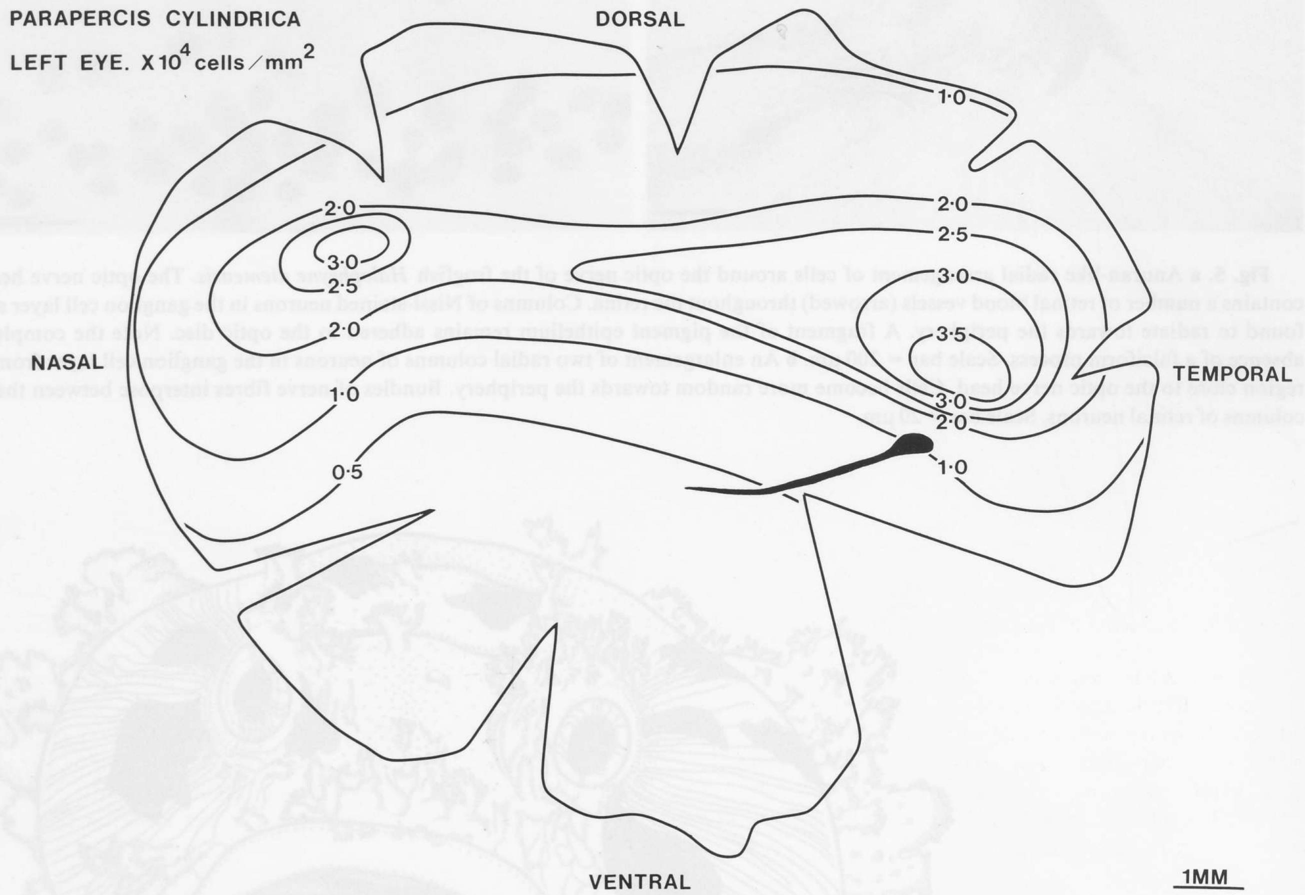
PARAPERCIS CYLINDRICA
LEFT EYE. X10⁴ cells/mm²
DORSAL
NASAL
TEMPORAL
VENTRAL
1·0
2·0
2·5
3·0
3·5
0·5
1MM

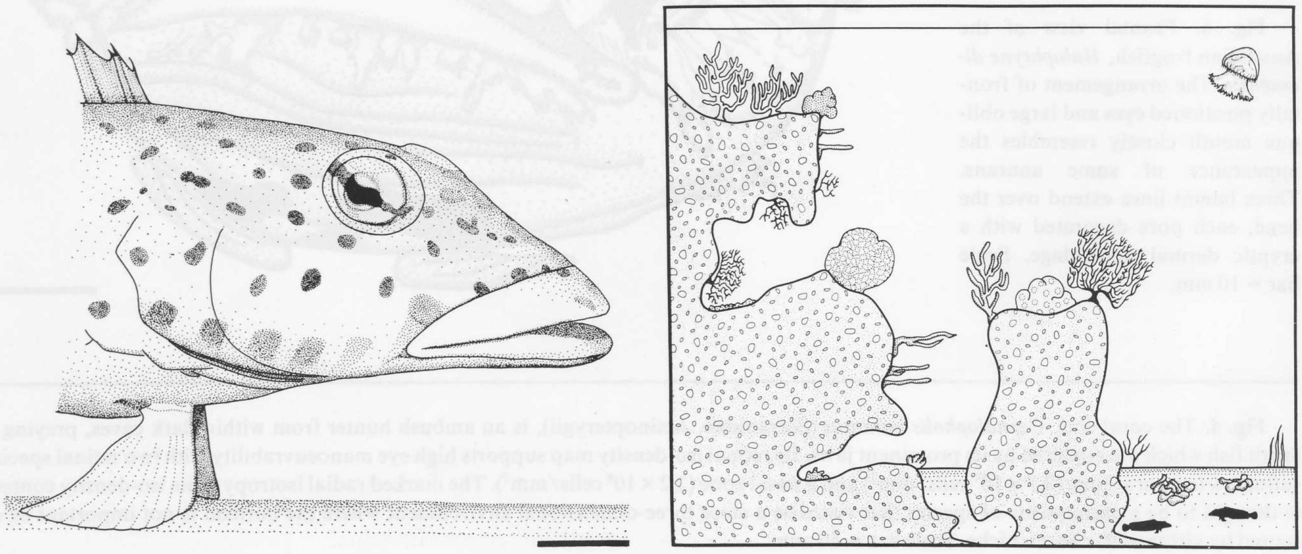

7

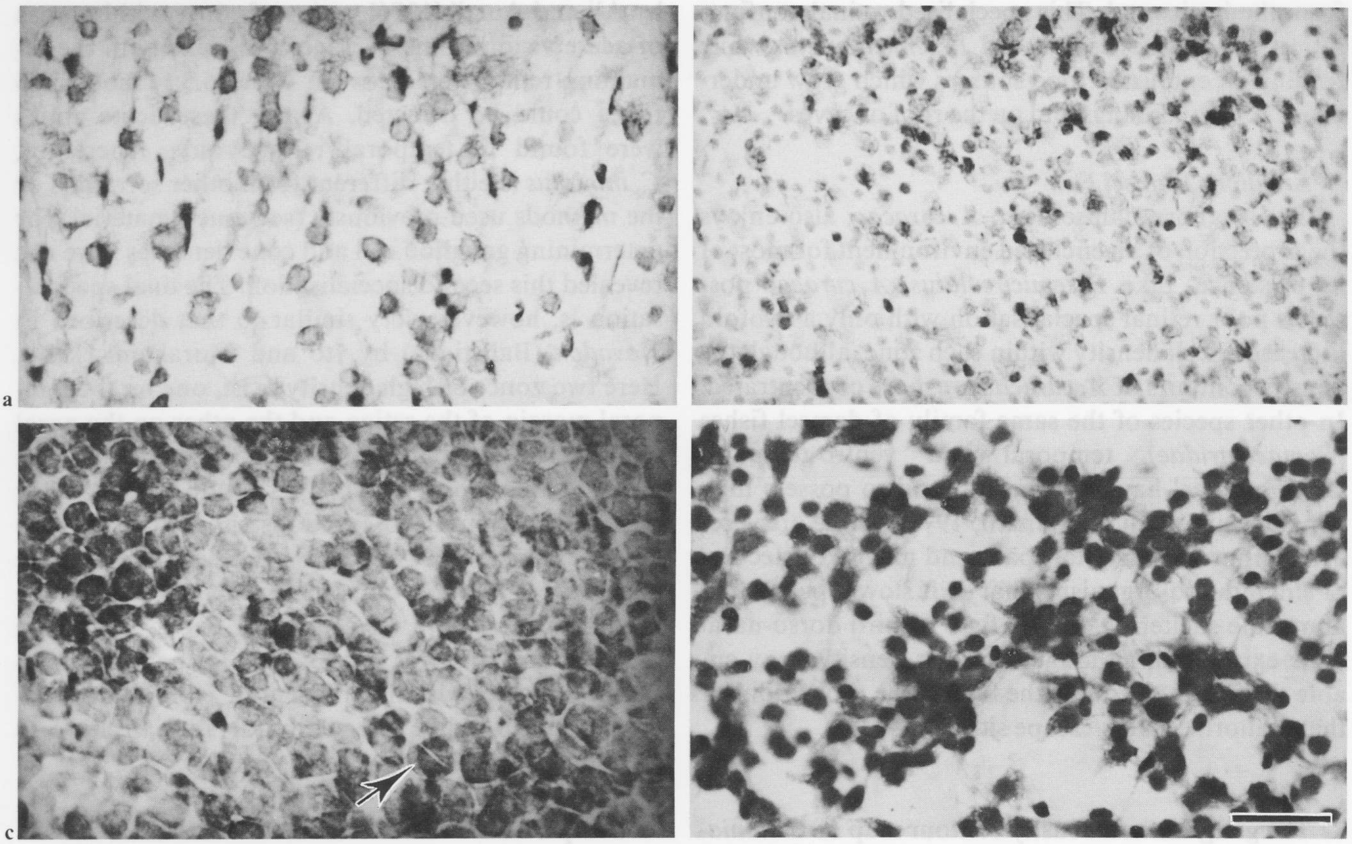

Fig. 8. Nissl-stained cells within the ganglion cell layer of the retina in four species of teleosts. The dorso-nasal zone of *A. curacao* (**a**) possesses a low density of ganglion cells with large diameter soma whereas the dorso-nasal zone of *P. cylindrica* (**b**) possesses a higher density of cells with smaller diameter soma. To compare the variation in soma size and packing, the ganglion cell layer of the blue tusk fish *Choerodon albigena* (**c**) [Collin and Pettigrew, 1988] has been included to emphasize the very high density of large, tightly packed cells that exists in the area centralis of some teleosts. The ganglion cell layer is stratified into two sublaminae where some cells overlie each other (arrowed). The cells of *C. miniatus* (**d**) reveal fine arborisations from densely stained soma which are randomly clumped within a temporal zone of the retina. Scale bar = 20 μm.

Fig. 7. The weever fish, *Parapercis cylindrica* (Parapercidae, Actinopterygii), possesses a dual retinal specialisation illustrated by a concentration of iso-density contours in two zones mediated by a weak band of increased cell density. The functions of a temporal zone (4.0×10^4 cells/mm^2) and a nasal zone (3.4×10^4 cells/mm^2) are emphasized by the highly moveable eyes which often move independent of one another. This small carnivore, supported by extended pectoral fins, surveys its sand or coral rubble habitat for small fish and crustaceans, often viewing its panoramic environment with independent, repeated eye movements. Each eye with its frontally tapered pupil fixates on prey within its binocular field. This species survives in fairly open water areas although it is a substrate feeder with a low profile and the horizon is relatively insignificant in its visual environment. Its escape response comprises flight into the nearest cave or under a coral overhang. Scale bar = 20 mm.

ering the likelihood that future study will reveal that only a fraction of these cells are ganglion cells sending an axon into the optic nerve.

Blue Angel Fish

The eyes of *Pomacanthus* sp. are highly movable and can swivel either frontally, for fixation of prey, or caudally, to perceive the approach of potential predators. The pupil is frontally tapered. However, due to the species' enclosed environment and the sessile nature of its food source, there may be little need for increased retinal specialisation. Radial isodensity contours are pronounced but form a weak band of specialisation inclined approximately 30° dorsal

from the horizontal. This specialised retinal configuration of acute zones may aid *P. semicirculatus* while feeding on encrusted food sources which grow underneath coral overhangs and on the roof of caves.

Staghorn Damsel Fish

The staghorn damsel fish, *A. curacao,* also enjoys the protection of an enclosed environment for most of its existence. Like *P. semicirculatus, A. curacao* possesses poor retinal specialisation with only a twofold increase of cell density within each zone, although the density contours of *A. curacao* are more concentrated. In other species of the same family of damsel fishes *(Pomacentridae),* temporal and ventro-temporal 'areae retinae' have also been found to possess high cone densities [Ali and Anctil, 1976]. These areas receive input from frontal space and may aid detection of small micro-organisms that drift slowly past, often above the sheltering damsel fish. A third dorso-nasal zone exists and, although of lower density, may enable the fish to negotiate the finger-like projections of the staghorn coral in escape situations.

Coral Cod

The ganglion cell density contour map of *C. miniatus* reveals two specialisations, a temporal area and a nasal area (fig. 4). The highest density of cells within the temporal area is 4.7×10^4 cells/mm^2, which is over four times the density of cells that lie over three quarters of the retina. The density contours of this 'enclosed habitat' species reveal a concentric configuration where the temporal area lies very close to the margin of the retina. The nasal area also occurs on the margin of the retina, and these two zones of high visual acuity are arranged on either end of the horizontal meridian and subtend an angle of 140°. The serranid eye may use these spatially separated retinal zones for monocular, as observed behaviourally for *C. miniatus,* and binocular fixation. This is obviously an advantage to a predator which must rely on catching fast-moving prey.

In another serranid, the kelp bass, *Paralabrax,* an acute temporal zone of approximately 3.3×10^4 cells/mm^2 has been found [Schwassmann, 1968]. This foveal region is comparable to that in *C. miniatus* and exhibits a 12-fold increase in ganglion cell density. However, no area centralis in the nasal retina has been observed. Cone density also provides an index of the resolving power of the retina, and in two Japanese serranids [Tamura, 1957] and another described by Ali and Anctil [1976], ratios of cones within areas of acute vision to the average cone density in the remaining retina were between 4 and 6.5:1, though no fovea could be detected. Again, these acute zones were found in temporal retinae only. Therefore, *C. miniatus* is either different from other serranids or the methods used previously (sectioned material) for determining ganglion cell and cone densities have not revealed this second specialisation. The dual specialisation is, however, very similar to that described in *Navodon* (Balistidae) by Ito and Murakami [1984]. Here two zones of high density exist, one on the temporal margin of the retina and the other on the nasal margin. This configuration has also been described in a frog, *Heleioporus eyrei,* by Dunlop and Beazley [1981]. Behavioural observations show that all three of these species exist in enclosed areas and can move backwards. *H. eyrei* burrows backwards to escape predation, and both teleosts swim backwards using their pectoral fins. It is possible that the nasal specialisation provides visual information from caudal eccentric space, while the temporal specialisation is presumably for feeding.

Sharp-Nosed Weever Fish

This fish has two widely separated zones of increased ganglion cell density, a dorso-temporal zone and a dorso-nasal zone, which subtend an angle of approximately 130° (fig. 7). The temporal zone is subserved by a rostral tapering of the pupil. The nasal zone is subserved by increased caudal eye mobility in conjunction with a weak horizontal streak extended across the retina, just dorsal of the horizontal meridian. It is possible that in this way *P. cylindrica* successfully utilises acute panoramic vision of its environment as an adaptation for detection of both prey and predators.

An almost identical combination of acute zones is found in quails [Budnik et al., 1984]. Both the temporal zone and the nasal zone have ganglion cell densities of $3.0–3.5 \times 10^4$ cells/mm^2. The total number of cells (130×10^4) is also similar to that in the weever fish (149×10^4, unpublished results). Behavioural correlations can also be made between these two species. The weever fish lives in areas of open sand and coral rubble and has eyes that are highly mobile and survey the horizon or water-sand interface. Hence the adjoining contours of higher ganglion cell density are oriented along the horizontal meridian. Quails are mostly terrestrial although they have only recently

been derived from stocks of predominantly aerial ancestors. Therefore, the arrangement of iso-density contours has evolved for a terrestrial habit of feeding [Podgett and Ivey, 1959], with circular configuration, while the horizontal component of higher ganglion cell density found in inhabitants of open horizons (such as the albatrosses) [Fite and Rosenfield-Wessels, 1975] has diminished.

The lack of retinal specialisation in species not solely reliant on visual cues for survival is also reflected centrally. Examination of the brains of these teleosts reveals a marked decrease in the relative size of the optic tectum compared to that in faster swimming species [Collin and Pettigrew, 1988]. This is particularly applicable to *H. diemensis* which has a reduced optic tectum and, indeed, a smaller brain than in faster swimming species of comparable size. Ito and Murakami [1984] also found that *Sebastiscus*, a sedentary scorpaenid without a horizontal streak, possessed a smaller optic tectum than an open water genus, *Navodon* (Balistidae).

Therefore, those species that frequent an 'enclosed' environment with an interrupted view of the sand-water horizon possess well-developed areae with a poorly-developed horizontal streak. Collin and Pettigrew [1988] investigate teleosts that possess even higher density areae with well-developed horizontal streaks.

Acknowledgements

This work was supported by grants to J.D. Pettigrew from the Australian Research Grants Scheme and the National Health and Medical Research Council. We thank Dr. I.D. Lawn and the staff of the Heron Island Research Station for their co-operation. Contribution Number 14 from the Neuroscience Laboratory.

References

Ali, M.A.; Anctil, M.: Retinas of fishes: An atlas (Springer, Berlin 1976).

Budnik, V.; Mpodozis, J.; Varela, F.J.; Maturana, H.R.: Regional specialization of the quail retina: Ganglion cell density and oil droplet distribution. Neurosci. Lett. *51:* 145–150 (1984).

Carcasson, R.H.: A field guide to the reef fishes of tropical Australia and the Indo-Pacific region (Collins, London 1977).

Collin, S.P.; Pettigrew, J.D.: Retinal topography in reef teleosts. II. Some species with prominent horizontal streaks and high density areae. Brain Behav. Evol. *31:* 283–295 (1988).

Dunlop, S.A.; Beazley, L.D.: Changing retinal ganglion cell distribution in the frog *Heleioporus eyrei*. J. comp. Neurol. *202:* 221–236 (1981).

Ebbesson, S.O.E.: Retinal projections in two teleost fishes (*Opsanus tau* and *Gymnothorax funebris*). An experimental study with silver impregnation methods. Brain Behav. Evol. *1:* 134–154 (1968).

Fite, K.V.; Rosenfield-Wessels, S.A.: A comparative study of deep avian foveas. Brain Behav. Evol. *12:* 97–115 (1975).

Frisen, L.; Frisen, M.: A simple relationship between the probability distribution of visual acuity and the density of retinal output channels. Acta ophthal. *54:* 437–443 (1976).

Fukuda, Y.: A three group classification of rat retinal ganglion cells: Histological and physiological studies. Brain Res. *119:* 327–344 (1977).

Grant, E.M.: Guide to fishes (Queensland Department of Harbours and Marine, Brisbane 1982).

Graydon, M.L.; Giorgi, P.P.: Topography of the retinal ganglion cell layer of *Xenopus*. J. Anat. *139:* 145–157 (1984).

Greenwood, P.H.; Rosen, D.E.; Weitzman, S.H.; Myers, G.S.: Phyletic studies of teleostean fishes, with a provisional classification of living forms. Bull. Am. Mus. nat. Hist. *131:* 339–455 (1966).

Gulliver, G.: Fovea centralis in the eye of the fish. J. Anat. Physiol., Lond. *2:* 12 (1868).

Hebel, R.: Distribution of retinal ganglion cells in five mammalian species (pig, sheep, ox, horse, dog). Anat. Embryol. *150:* 45–51 (1976).

Hughes, A.: Topographical relationships between the anatomy and physiology of the rabbit visual system. Documenta Ophth. *30:* 33–159 (1971).

Hughes, A.: A comparison of retinal ganglion cell topography in the plains and tree kangaroo. J. Physiol., Lond. *244:* 61–63 (1974).

Hughes, A.: The topography of vision in mammals; in Crescitelli, Handbook of sensory physiology, vol. 7 (Springer, Berlin 1977).

Hughes, A.: New perspectives in retinal organisation; in Osbourne, Chader, Progress in retinal research, vol. 4, pp. 243–313 (Pergamon Press, Oxford 1985).

Ito, H.; Murakami, T.: Retinal ganglion cells in two teleost species, *Sebastiscus marmoratus* and *Navodon modestus*. J. comp. Neurol. *229:* 80–96 (1984).

Kahmann, H.: Über das Vorkommen einer Fovea centralis im Knochenfischauge. Zool. Anz. *106:* 49–55 (1934).

Kahmann, H.: Über das foveale Sehen der Wirbeltiere. II. Gesichtsfeld und Fovea centralis. Sitz. Ges. naturf. Freunde, pp. 361–376 (1935).

Perry, V.H.; Cowey, A.: A sensitive period for ganglion cell degeneration and the formation of aberrant retino-fugal connections following tectal lesions in rats. Neuroscience *7:* 583–594 (1982).

Podgett, A.A.; Ivey, E.D.: Coturnix quail as a laboratory research animal. Science *129:* 267–268 (1959).

Provis, J.M.: The distribution and size of ganglion cells in the retina of the pigmented rabbit: A quantitative analysis. J. comp. Neurol. *185:* 121–138 (1979).

Rodieck, R.W.: The vertebrate retina: Principles of structure and function (Freeman, San Francisco 1973).

Rolls, E.T.; Cowey, A.: Topography of the retina and striate cortex and its relationship to visual activity in rhesus monkeys and squirrel monkeys. Exp. Brain Res. *10:* 298–310 (1970).

Schwassmann, H.O.: Visual projection upon the optic tectum in foveate marine teleosts. Vision Res. *8:* 1337–1348 (1968).
Slonaker, J.R.: A comparative study of the area of acute vision in vertebrates. J. Morph. *13:* 445–492 (1897).
Stone, J.: A quantitative analysis of the distribution of ganglion cells in the cat's retina. J. comp. Neurol. *124:* 337–352 (1965).
Stone, J.; Johnston, E.: The topography of primate retina: A study of the human, bushbaby, and new- and old-world monkeys. J. comp. Neurol. *196:* 205–223 (1981).
Tamura, T.: A study of visual perception in fish, especially on resolving power and accommodation. Bull. Jap. Soc. Sci. Fish *22:* 536–557 (1957).
Wilson, M.A.: Optic nerve fibre counts and retinal ganglion cell counts during development of *Xenopus laevis* (Daudin). Q. Jl exp. Physiol. *56:* 83–91 (1971).
Yamanouchi, T.: The visual acuity of the coral fish, *Microcanthus strigatus* (Cuvier Valenciennes). Publ. Seto. Mar. Biol. Lab. *5:* 133–156 (1956).

Shaun P. Collin
Department of Physiology and Pharmacology
University of Queensland
St. Lucia, QLD 4067 (Australia)
