# Supplementary material for: A Comparison of Spatial Analysis Methods for the Construction of Topographic Maps of Retinal Cell Density
Source: PLoS One. 2014 Apr 18;9(4):e93485. doi: 10.1371/journal.pone.0093485 (PMC3998654; doi:10.1371/journal.pone.0093485)
Supplement: File S7 — Collin and Partridge 1996.pdf. Paper where the original map of the smooth-head fish, Conocara murrayi. Collin SP, Partridge JC (1996) Retinal specializations in the eyes of deep-sea teleosts. Journal of Fish Biology 49: 157–174. (PDF) [file pone.0093485.s007.pdf]

Journal of Fish Biology (1996) **49** (Supplement A), 157–174

FISH VISION

Retinal specializations in the eyes of deep-sea teleosts

S. P. COLLIN* AND J. C. PARTRIDGE†

**Marine Neurobiology Laboratory, Department of Zoology, University of Western Australia, Nedlands, 6907 Western Australia, Australia and †School of Biological Sciences, University of Bristol, Woodland Rd, Bristol BS8 1UG, U.K.*

Although living beyond the penetration limits of sunlight, many deep-sea teleosts possess large eyes, lenses capable of accommodation, and various adaptations for increasing sensitivity and extending their visual field. However, little is known of the extent of the visual field and whether the spatial resolving power of the eye is constant across the retina. In order to examine whether these fish are specialized for acute vision in particular regions of their visual field, retinal wholemounts were used to examine the regional differences in the density of retinal ganglion cells in 16 species from different depths, habitats and photic zones. It was found that the retinal ganglion cell topography changes markedly across the retina with a density range of 6·3–50·6 × 10^3 cells mm^{-2} in a pattern unique to each species. A number of mesopelagic species including the lanternfishes, *Lampanyctus macdonaldi* and *Myctophum punctatum* possess a concentric increase in cell density towards the retinal margins, putatively enhancing peripheral vision. Three tubular-eyed species including *Scopelarchus michaelsarsi* possess an area centralis in the centro-lateral region of the main retina supporting the premise that this specialization receives a focused image. Some benthic species such as the smoothhead *Rouleina attrita* and the searsid *Searsia koefoedi* also boast a structural specialization or fovea in temporal retina with centro-peripheral cell gradients exceeding 30 : 1. Benthic species such as the tripodfish *Bathypterois dubius* possess two regional increases in ganglion cell density or areae centrales, one nasal and the other temporal, thereby increasing spatial resolving power in the caudal and rostral visual fields, respectively. These quantitative analyses suggest that deep-sea fishes, like their shallow-water counterparts, also use a specific region of their visual field for acute vision. This may be an advantage for the detection and localization of bioluminescent light sources.

© 1996 The Fisheries Society of the British Isles

Key words: teleosts; vision; retina; area centralis; fovea; ganglion cells.

INTRODUCTION

Vision plays an enormous role in the survival of many species of teleosts that live in shallow, well-lit environments. Specializations, such as foveae (Schwassmann, 1968; Collin & Collin, 1988*a*,*b*) and increases in photoreceptor (O'Connell, 1963; Ahlbert, 1976; Zaunreiter *et al.*, 1991) and ganglion cell (Collin & Pettigrew, 1988*a*,*b*; Collin, 1989) density in specific regions of the retina, mediate acute vision by higher sampling of a discrete region of each species' visual world. In addition to the few studies on teleosts (Collin & Pettigrew, 1988*c*), studies of retinal ganglion cell topography, as revealed in wholemount, have received particular attention in birds (Reymond, 1985) and mammals (Pettigrew *et al.*, 1988) also, where such studies have predicted successfully the limits of visual acuity in areas of highest ganglion cell density. In regions of highest acuity (areae centrales), there is a close relationship between the photoreceptors and the

*Tel.: +61 9 380 3233; fax: +61 9 380 1029; email: scollin@uniwa.uwa.edu.au

0022–1112/96/49A157+18 $25.00/0 © 1996 The Fisheries Society of the British Isles

ganglion cells where the convergence of the photoreceptors and the higher order ganglion cells is low in comparison with other regions of the retina. Photopic visual acuity depends on the solid angle viewed by the eye and the photoreceptor spacing (Fernald, 1988) and, therefore, the least separable visual angle approximates the angle subtended by one ganglion cell, for each ganglion cell possesses a separate conductive axon which projects to the visual centres of the brain via the optic nerve. Since the retinal ganglion cell mosaic provides the only link between the eye and behavioural output, the spatial distribution of the retinal ganglion cells places an upper limit on the spatial resolving power of the whole animal (Collin & Pettigrew, 1989).

The location and arrangement of these retinal specializations correlate closely with the symmetry of each species' perceived world (Collin & Pettigrew, 1988*a,b*). Species with a predominantly clear view of their horizon (sand–water or air–water interface) possess a marked increase in the density of retinal neurons across the retinal meridian in the form of a horizontal or visual streak (Munk, 1970; Collin & Pettigrew, 1988*b*). These species possess a panoramic view of their environment and mediate acute vision without the saccadic eye movements necessary for an area centralis (Hughes, 1977), thereby providing a lower threshold for the perception of movement and the signalling of either prey or predator. In contrast, teleosts with an interrupted view of their horizon do not possess an elongated increase of retinal neurons but a concentric arrangement of retinal iso-density contours. These species live in enclosed or more three-dimensional environments with an interrupted view of their horizon, and often possess a high degree of eye mobility and a temporal area centralis subtending a frontal binocular field.

In contrast, little is known of the retinal specializations of deep-sea teleosts which have evolved under remarkable selection pressures in a world characterized by extremely dim sunlight (visually irrelevant below *c.* 1000 m, Denton, 1990) and bioluminescence. In fact, the extreme environmental constraints for the detection of prey, predators and mates have, in some species, elicited radical changes in the structure of the eye. Some eyes have become tubular to take advantage of the down-welling sunlight but at the expense of a large visual field (i.e. *Argyropelecus aculeatus* Valenciennes, 1849 and *Scopelarchus michaelsarsi* Koefoed, 1955), while others have degenerated (i.e. *Sciadonus kullenbergi* (Nybelin, 1957) and *Bathymicrops regis* Hjort & Koefoed, 1912, Munk, 1964, 1965), these animals relying on other sensory modalities. In the deep sea, prey, predators and mates are signalled by dim, distant point sources of light such as bioluminescence and, therefore, it has been assumed that optimizing sensitivity (with high summation ratios between photoreceptors and ganglion cells) is of prime importance in the survival of these species.

This is exemplified by the finding of mesopelagic and benthopelagic deep-sea teleost retinae which possess high densities of rods, either elongated (i.e. in *Diretmus argenteus* Johnson, 1863, Munk, 1966*a*), arranged in multiple banks [i.e. in *Bajacalifornia megalops* Lütken, 1898 (formerly *Bajacalifornia drakei* Beebe, 1929), Locket, 1985; Denton & Locket, 1989; Fröhlich *et al.*, 1995] or grouped to form ‘ macroreceptors ’ (i.e. in *Scopelarchus guntheri* Alcock, 1896, Locket, 1971*a*, 1977). However, the finding of a fovea in *Bathylagus euryops* Goode & Bean, 1896 (formerly *Bathylagus benedicti* Goode & Bean, 1896)

(Vilter, 1954), *Platytroctes apus* Günther, 1987 (formerly *Platytroctes procerus* (Locket, 1971*b*), *Bathylaco nigricans* Goode & Bean, 1896 (Munk, 1968) and a number of alepocephalids (Collin *et al.*, 1994) suggests that ganglion cell densities may also increase in these specialized zones as they do in other shallow-water teleosts (Schwassmann, 1968; Collin & Collin, 1988*a*,*b*), thereby reducing the summation ratio and increasing the resolving power.

Other clues exist to support the premise that deep-sea teleosts are utilizing specific regions of their visual field for acute vision. These include the rostral tapering of the pupil and a binocular sighting groove in the snout of most alepocephalid and searsid species to increase the amount of binocular field subtended by the temporal fovea (Collin *et al.*, 1994), the evolution of tubular eyes (Munk, 1966*b*; Locket, 1977), and the development of specialized feeding mechanisms for the capture of prey (i.e. in *Stylephorus chordatus* Shaw, 1791, Pietsch, 1978).

The technique utilized in this study for investigating the changing role vision plays in various deep-sea environments is the examination of retinal ganglion cell topography. This wholemount method enables the entire retina to be examined and an assessment made of the limits of spatial resolving power of the retina in a range of species to gain some insight into their feeding strategies and visual capabilities.

MATERIALS AND METHODS

Sixteen species of teleosts (at least two specimens of each) were collected on two scientific expeditions on board the RRS ‘Challenger’ in 1992 (Cruise No. 94) and the RRS ‘Discovery’ Cruise in 1993 (Cruise No. 204). The Challenger cruise was in the vicinity of 49–51° N (latitude) and 110–150° W (longitude) over the Globan Spur and the Porcupine Sea Bight, and the Discovery cruise was in the vicinity of 18–20° N (latitude) and 19–21° W (longitude) over the Madeira Abyssal Plain. All species were captured with rectangular mid-water trawling nets (RMT 1+8) with or without closing cod-ends (CCE) or semi-balloon otter trawling nets (OTSB 14) at depths between 100 and 2500 m.

For all specimens, sampling was carried out according to the ethical guidelines of the National Health and Medical Research Council of Australia. Most animals were dead when the trawl was brought on deck but some animals were killed by immersion in an overdose of tricaine methane sulphonate (MS 222, 1 : 2000) in sea water, after which the eyes were excised. Both the left and right eyes of each animal were immersion-fixed in 4% paraformaldehyde in 0·1 M phosphate buffer or in a mixture of 2·5% glutaraldehyde and 2% paraformaldehyde in 0·1 M phosphate buffer (pH 7·4). Those eyes destined for morphological analyses were postfixed overnight and either remained whole or were cut into small pieces and washed in phosphate buffer before being embedded in Historesin (Reichert-Jung). One- to 2-μm sections were cut using an LKB Nova motorized ultramicrotome and glass knives. Transverse sections were stained with toluidine blue, dehydrated and coverslipped for analysis with an Olympus BH-2 compound light microscope. Photographs were taken on Technical Pan (50ASA) black-and-white film.

COUNTING CELLS IN RETINAL WHOLEMOUNT

The retina of the eye not used for sectioning in each species was wholemounted. Each eye was immersion-fixed in 4% paraformaldehyde in phosphate buffer (0·1 M) for 40 min. Following fixation, and while immersed in phosphate buffer, the entire retina of each eye was carefully dissected free of its scleral eye-cup and retinal pigment epithelium. The

retina was then wholemounted, ganglion cell layer uppermost, on a 5% gelatinized slide by making a series of slits in the periphery of the retina. The orientation of each retina was noted and confirmed by making a small incision at the dorsal and nasal retinal margins. Each retina was flattened by sandwiching it between its slide of attachment and two pieces of filter paper (Whatman Nos. 1 and 50 both soaked in a 1 : 1 mixture of formaldehyde and absolute alcohol), a second glass slide and a brass weight. After drying overnight, the retinal ganglion cell layer was stained for Nissl substance in cold 0·05% cresyl violet (in acetate buffer, pH 4·3) for 2–4 min. Dehydration and clearing preceded coverslipping in DPX.

Densities of stained cells or cell nuclei within the ganglion cell layer were determined for both left and right eyes (in different individuals of the same species), although the topography of only one eye of each species is presented. The counting protocol closely follows that of Collin & Pettigrew (1988*a*). Briefly, numbers of cells/nuclei within each microscope field were counted routinely every 0·5 mm across the retina, but, in areas of higher density, cell numbers were counted every 0·1 mm on the retina. These numbers were then converted to cells mm^{-2}. In this way, up to 350 areas per retina were sampled, allowing determinations of small fluctuations in cell density. Iso-density contours were constructed by interpolation between similar values of retinal ganglion cell densities. No allowance was made for retinal shrinkage which is known to be less than 2% for this technique (Hughes, 1975; Mednick & Springer, 1988).

All recognizable neural elements lying within the retinal ganglion cell layer were counted. If cellular elements were located clearly between the optic nerve fibre layer and the inner plexiform layer and had some Nissl substance in their cytoplasm, they were counted, independent of size. Therefore, although glial cells, distinguished by their smaller size, cigar shape and dark staining (Hughes, 1985), were not included in cell counts, the densities (and therefore spatial resolving powers, see below) calculated represent upper limits which may have to be revised downwards when data from retrograde transport studies separate the ganglion cells from displaced amacrine cells (known to lie within the ganglion cell layer) conclusively. However, in other studies of teleost retinae, the major contribution of these non-ganglion cells has been found to lie in non-specialized regions of the retina with the topography and the peak densities of the true population of ganglion cells remaining relatively unchanged, even with their inclusion in cell counts (Collin & Pettigrew, 1988*c*).

SPATIAL RESOLVING POWER

The peak spatial resolving power was calculated for the maximum cell density in the retina of each species according to the method of Collin & Pettigrew (1989), which calculates the angular subtense of the ganglion cells projected into visual space by a lens obeying Matthiessen's ratio (Matthiessen, 1880). The software package NIH Image 1·55 was used in conjunction with a Power Macintosh 7100 to determine the retinal area and the total number of cells within the ganglion cell layer, by measuring the area bounded by each iso-density contour and multiplying each area by its average cell density.

RESULTS

All species examined revealed a non-uniform distribution of cells within the retinal ganglion cell layer. This non-uniformity was revealed as a localized increase in cell density, or area centralis, or as a concentric increase in cell density towards the peripheral retinal margins. In some species, an area centralis was also associated with a foveal depression.

In the bigfin pearleye *Scopelarchus michaelsarsi*, and the hatchetfishes *Argyropelecus sladeni* Regan, 1908 and *A. aculeatus*, all of which possess tubular eyes, the retina is divided into two regions; a main retina subserving the dorsal binocular field and the accessory retina subserving the monocular dorso-lateral

visual field. The main retina of each species possesses a centro-lateral area centralis between 30·0 and 50·6 × 10^3 ganglion cells mm^{-2} packed into the ganglion cell layer up to four cells deep [Table I, Figs 1(a,c) and 2(a),(c),(d)]. The accessory retina is relatively unspecialized accounting for the high centro-peripheral density gradients up to 37 : 1.

The mesopelagic searsid, *Searsia koefoedi* Parr, 1937 and the bluntsnout smoothhead *Xenodermichthyes copei* Gill, 1884 both reveal a peak density of approximately 24 × 10^3 ganglion cells mm^{-2} near the temporal margin of the retina with a second nasal area in *X. copei* of over 9 × 10^3 cells mm^{-2} [Table I; Figs 2(b), 3 and 4(c)]. The optic nerve and falciform process in each species is elongated and extends horizontally from the ventro-nasal margin to the temporal retina. The temporal area centralis of each species is also associated with a steep-sided depression or convexiclivate fovea [Fig. 3(c)]. Peak densities of ganglion cells are located in the thick perifoveal region while the cell density drops off to zero in the centre of the depression underlying a layer comprised of radial fibres [Fig. 3(b)]. In this region, the rods are elongated (over 200 µm in length) and often protrude into the sclera to form a fovea externa. In a wholemount view of the retina of *X. copei*, this region of radial fibre origin appears to show a rosette pattern emanating from the centre of the foveal depression [Fig. 3(a)].

The deep-sea bass *Howella sherborni* (Norman, 1920) also possesses a temporal fovea with multiple banks of rods and a ganglion cell density of 24·5 × 10^3 cells mm^{-2} [Table I; Fig. 4(a)]. However, this region of high density extends over much of the temporal peripheral retina subtending a large area of frontal binocular visual space.

Other mesopelagic species reveal a concentric increase in ganglion cell density towards the retinal margin. Both lanternfishes *Myctophum punctatum* Rafinesque, 1810 [peripheral peak of 19·4 × 10^3 cells mm^{-2}, Fig. 4(b)] and *Lampanyctus macdonaldi* (Goode & Bean, 1896) [peripheral peak of 17·1 × 10^3 cells mm^{-2}, Figs 1(b) and 4(d)] and the abyssopelagic, sawtoothed eel *Serrivomer beani* Gill & Ryder, 1883 [peripheral peak of 12·5 × 10^3 cells mm^{-2}, Fig. 6(c)] show an unspecialized central retina with higher spatial resolving power in peripheral retina.

The engybenthic species living and feeding on, or just above, the bottom all show a similar topographic retinal pattern. All species examined possess large eyes, a rostral tapering of the pupil or aphakic gap and a temporal fovea. Foveal specialization is variable with the degree of invagination, inner retinal layer displacement and the length of the underlying photoreceptors different in each species. Highest peak densities were found in the smallscale smoothhead *Bathytroctes microlepis* Günther, 1878 [30·8 × 10^3 cells mm^{-2}, Fig. 5(c)] while both Risso's smoothhead *Alepocephalus rostratus* (Risso, 1820) (temporal peak of 24·6 × 10^3 cells mm^{-2}) and the softskin smoothhead *Rouleina attrita* (Vaillant, 1888) [temporal peak of 27·4 × 10^3 cells mm^{-2}, Fig. 1(d)] possess a second area centralis in the nasal retina of 12·6 × 10^3 cells mm^{-2} and 7·8 × 10^3 cells mm^{-2}, respectively [Fig. 5(b),(d)]. The temporal specialization in *R. attrita* does not appear to be in the form of a foveal depression but a localized increase in retinal ganglion cell density [Fig. 5(d)] in conjunction with an elongation of rods and a scleral bulge or fovea externa. Although the retina of the smoothhead

TABLE I. Summary of data collected on the 16 species of teleosts examined in this study arranged in approximate order of their depth in the water column

Species	Family	Depth recorded (m)	Depth caught (m)	Habitat	Body size (mm)	Peak RGC density	Position of AC	Peak density gradient	Total RGC	Lens diameter (mm)	Spatial resolution
Myctophum punctatum	Myctophidae	700–1000	750	Mesopelagic	96; 85	19·4	Increases peripherally	3·2 : 1	9·05	NR	NK
Lampanyctus macdonaldi	Myctophidae	550–1100	1100	Mesopelagic	165; 145	17·1	Increases peripherally	2·4 : 1	9·31	NR	NK
Xenodermichthyes copei	Alepocephalidae	100–1000	2450	Mesopelagic	145; 140	24·3<br>9·2	Temporal<br>Dorso-nasal	9·3 : 1	8·79	1·84	6·4; 9·4
Howella sherborni	Moronidae	25–300	NR	Mesopelagic	NR	24·5	Temporal	2·7 : 1	14·10	NR	NK
Scopelarchus michaelsarsi	Scopelarchidae	256–500	250–310	Mesopelagic	66; 54	46·6	Centro-lateral	24·5 : 1	2·37	2·25	5·6; 10·7
Argyropelecus aculeatus	Sternoptychidae	100–600	NR	Mesopelagic	NR	50·6	Centro-lateral	19·5 : 1	6·82	NR	NK
Argyropelecus sladeni	Sternoptychidae	50–800	180–220	Mesopelagic	60; 48	30·0	Centro-lateral	37·5 : 1	3·86	3·05	6; 10
Searsia koefoedi	Searsiidae	450–1500	430–500	Mesopelagic	157; 138	23·8	Temporal	14 : 1	5·30	4·25	7·4; 8·1
Rouleina attrita	Alepocephalidae	1400–2100	1650	Engybenthic	350; 310	27·4<br>7·8	Temporal<br>Nasal	19·6 : 1	16·60	3·43	12·7; 4·7
Conocara murrayi	Alepocephalidae	1200–2600	2000	Engybenthic	534; 519	11·2	Temporal	37·3 : 1	7·30	NR	NK
Bathytroctes microlepis	Alepocephalidae	1100–2700	2000	Engybenthic/ Bathypelagic	255; 225	30·8	Temporal	22·0 : 1	15·03	3·48	13·5; 4·4
Alepocephalus rostratus	Alepocephalidae	300–3600	750	Engybenthic	NR; 140	24·6<br>12·6	Temporal<br>Nasal	10·7 : 1	14·21	3·36	11·9; 5·0
Synaphobranchus kaupi	Synaphobranchidae	400–4800	1200	Benthypelagic	370; NR	6·3	Ventral	4·2 : 1	1·96	NR	NK
Platytroctes apus	Searsiidae	385–>1000	1600–1800	Bathypelagic	148; 129	26·2<br>5·3	Temporal<br>Nasal	15·4 : 1	5·41	5·10	9·3; 6·5
Bathypterois dubius	Chlorophthalmidae	750–1950	1200	Benthic	170; 150	7·8<br>7·3	Temporal<br>Nasal	3·3 : 1	0·30	NR	NK
Serrivomer beani	Serrivomeridae	150–3000	960	Epipelagic/ Abyssopelagic	470; NR	12·5	Increases peripherally	2·5 : 1	2·36	NR	NK

Dimensions of body size (mm total length : mm standard length) and lens diameters (mm) are measured in fresh (unfixed) material. The recorded depth ranges and habitat of each species are from Whitehead *et al.* (1986) and all depths are given in m. Retinal ganglion cell (RGC) densities are $\times 10^3$ cells mm^{-2}. The total RGC numbers are $\times 10^5$ cells. The peak density gradient refers to the maximum gradient from the peak RGC density at the area centralis (AC) to the lowest RGC density over the retina. The spatial resolving power is calculated using Matthiessen's ratio (see Collin & Pettigrew, 1989) and is presented in cycles per degree; min of arc. NR, not recorded; NK, not known.

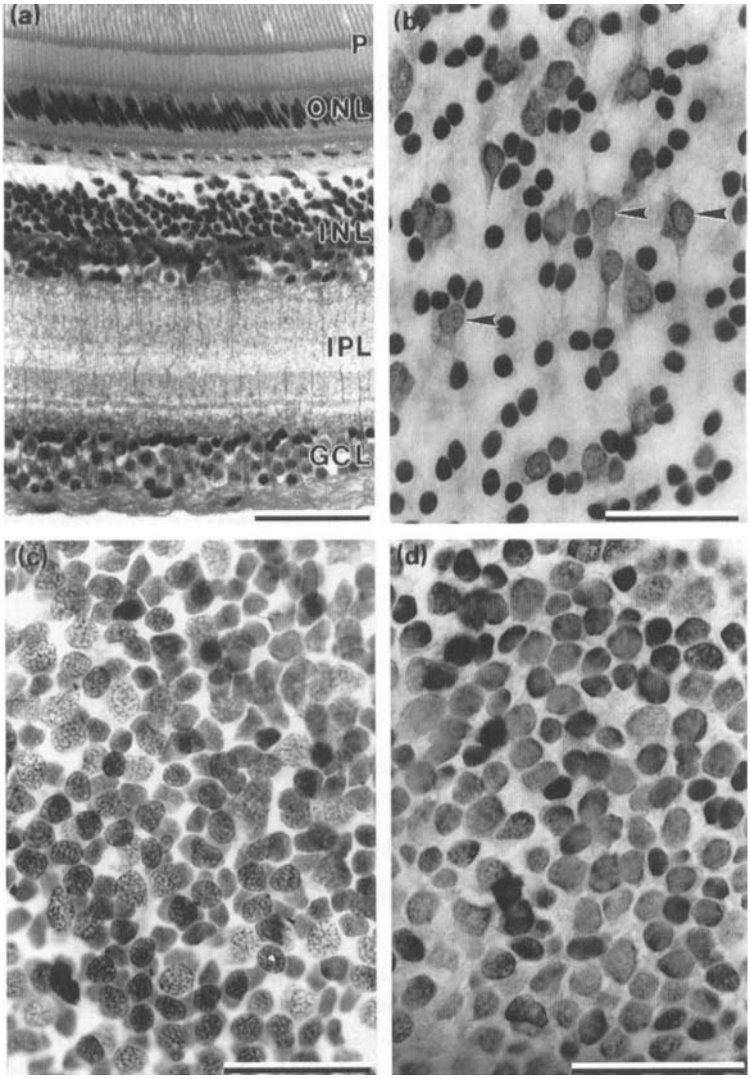

FIG. 1. (a) Transverse section (1 μm) of the main retina of *Scopelarchus michaelsarsi*. Note the large density of retinal ganglion cells in the ganglion cell layer (GCL) and thick differentiated inner plexiform layer (IPL). INL, inner nuclear layer; ONL, outer nuclear layer; P, photoreceptor layer. Scale bar 50 μm. (b) The ganglion cell layer in the peripheral retina of *Lampanyctus macdonaldi* viewed in wholemount showing a heterogeneous group of large (arrowed) and small soma. Scale bar 25 μm. (c) The arrangement and density of ganglion cells in the nasal area centralis of the main retina in *Argyropelecus aculeatus* viewed in wholemount. Scale bar 25 μm. (d) The close packing of ganglion cells in the specialized temporal retina of *Xenodermichthyes copei* viewed in retinal wholemount. Scale bar 25 μm.

Conocara murrayi (Koefoed, 1927) reveals the lowest density of ganglion cells in the temporal fovea ($11{\cdot}2 \times 10^3$ cells mm^{-2}), it possesses the highest temporo-peripheral gradient of any of the alepocephalids examined [37 : 1, Table I; Fig. 5(c)]. It is interesting to note the oblique orientation of the falciform process in *C. murrayi* compared to the other alepocephalids (Figs 4 and 5).

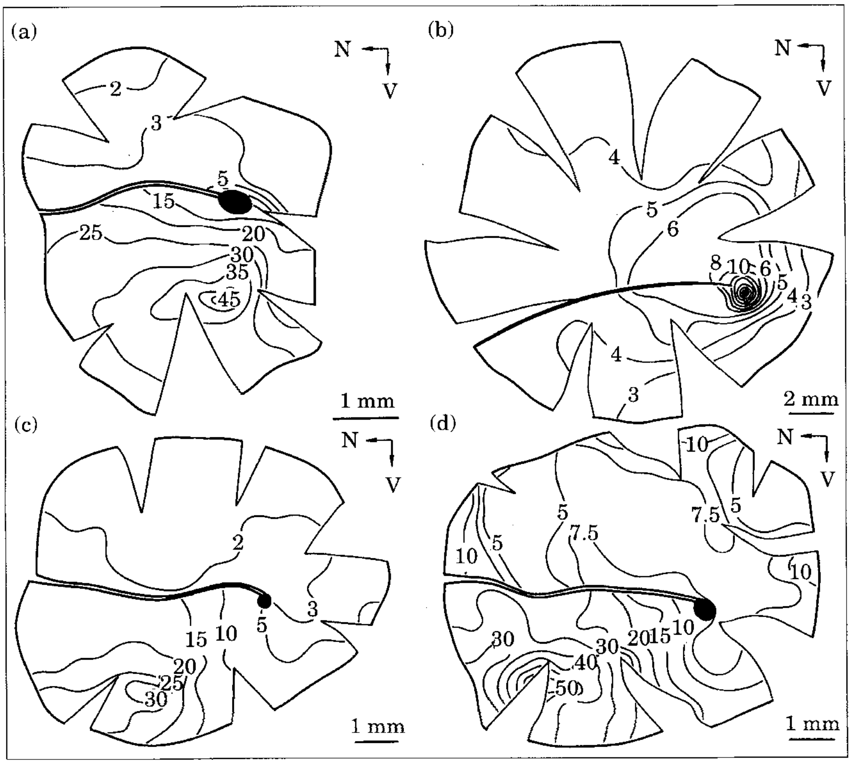

FIG. 2. Iso-density contour maps of the left eyes of four mesopelagic deep-sea species showing the topography of the retinal ganglion cell distribution. (a) *Scopelarchus michaelsarsi*. (b) *Searsia koefoedi*. (c) *Argyropelecus sladeni*. (d) *Argyropelecus aculeatus*. All densities are $\times 10^3$ cells mm^{-2} and the peak density in (b) is 23 800 cells mm^{-2}. The areae centrales in the tubular-eyed species (a, c, d) are located in the main retina, ventral of the horizontal falciform process. The optic nerve head and falciform processes are depicted in black. N, nasal; V, ventral.

The bathypelagic species all show unique topographic distributions presumably adaptive to their ecological niche. The legless searsid *Platytroctes apus* Günther, 1878, like *S. koefoedi*, possesses a temporal fovea with a peak of $26{\cdot}2 \times 10^3$ cells mm^{-2} and a nasal area centralis of $5{\cdot}3 \times 10^3$ cells mm^{-2} [Fig. 6(a)]. In contrast, the arrowtooth eel *Synaphobranchus kaupi* Johnsen, 1862 possesses a relatively unspecialized retina except for a slight increase in ganglion cell density along the ventral margin from less than 3·0 to $6{\cdot}3 \times 10^3$ cells mm^{-2} [Fig. 6(b)]. The eyes of the benthic tripodfish *Bathypterois dubius* Vaillant, 1888 elicit a yellow eye shine and contain two retinal specializations; a temporal area centralis of $7{\cdot}8 \times 10^3$ cells mm^{-2} and a nasal area centralis of $7{\cdot}3 \times 10^3$ cells mm^{-2} [Fig. 6(d)].

The total number of ganglion cells in each species varies from $0{\cdot}3 \times 10^5$ cells in *B. dubius* to $16{\cdot}6 \times 10^5$ cells in *R. attrita* (Table I).

DISCUSSION

Topographic studies of the retinal ganglion cell layer of 16 species of deep-sea teleosts from various habitats and photic zones reveal a non-uniform distribution

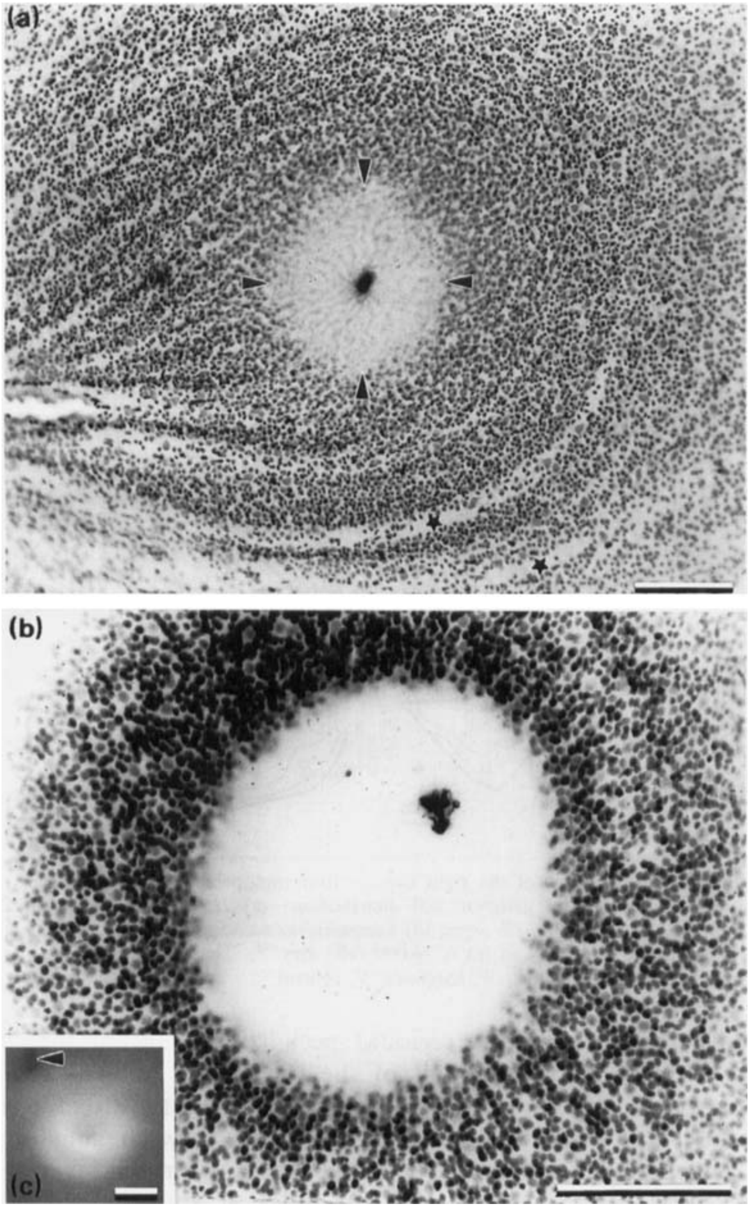

FIG. 3. (a) Low magnification wholemount view of the elevated temporal specialization in the retina of *Xenodermichthyes copei* following differential staining of the retinal ganglion cell layer. The arrowheads bound a thick rosette of radial fibres overlying a perifoveal region of peak ganglion cell density. The stars depict the tracts of vitreal blood vessels which emanate from the falciform process and surround this specialization. Scale bar 100 μm. (b). Close up of the temporal specialization of *Searsia koefoedi* showing the increase in ganglion cell density towards the foveal rim. Scale bar 50 μm. (c) Fundus view of the temporal specialization of *S. koefoedi* in an unfixed eye showing the raised foveal pit below the falciform process (arrowhead). Scale bar 150 μm.

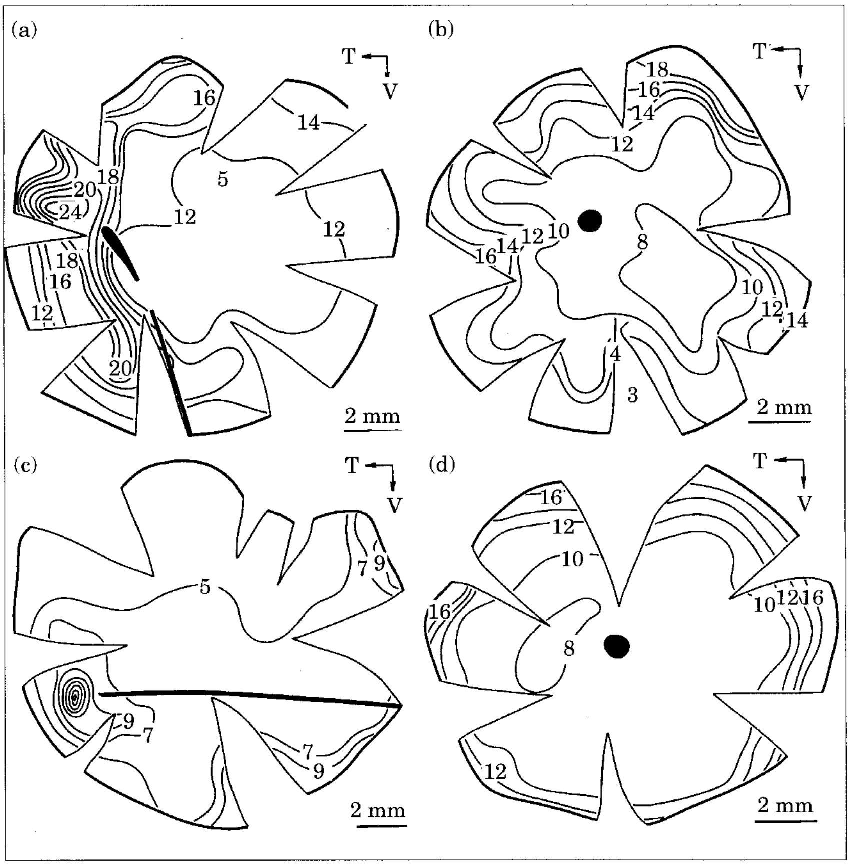

FIG. 4. Iso-density contour maps of the right eyes of four mesopelagic deep-sea species showing the topography of the retinal ganglion cell distribution. (a) *Howella sherborni*. (b) *Myctophum punctatum*. (c) *Xenodermichthyes copei*. (d) *Lampanyctus macdonaldi*. All densities are $\times 10^3$ cells mm^{-2} and the peak density in (c) is 24 000 cells mm^{-2}. The optic nerve head and falciform processes are depicted in black. T, temporal; V, ventral.

in all animals. Single or multiple regional specializations such as areae centrales and foveae subtend discrete regions of the visual field and, in a number of mesopelagic species, cell density increases concentrically towards the periphery.

In this study, no attempt was made to differentiate between ganglion cells and displaced amacrine cells known to lie within the ganglion cell layer in shallow-water teleosts (Ito & Murakami, 1984; Mednick & Springer, 1988; Collin & Pettigrew, 1988*c*). Therefore, the spatial resolving powers listed in Table I are upper limits which need to be revised downwards when retrograde labelling studies from the optic nerve become available and differentiate between displaced amacrine and ganglion cell populations. However, retrograde labelling from the optic nerve with the carbocyanine dye DiI in *Scopelarchus michaelsarsi* (S. P. Collin, R. V. Hoskins & J. C. Partridge, unpubl. data) reveal that over 86% of the cells in the ganglion cell layer of a region near the centro-lateral area centralis (1·5 mm eccentricity) possess an axon joining the optic nerve. Even higher proportions (92%) of ganglion cells have been found in the area centralis after retrograde labelling in the blue tuskfish *Choerodon cyanodus* Richardson,

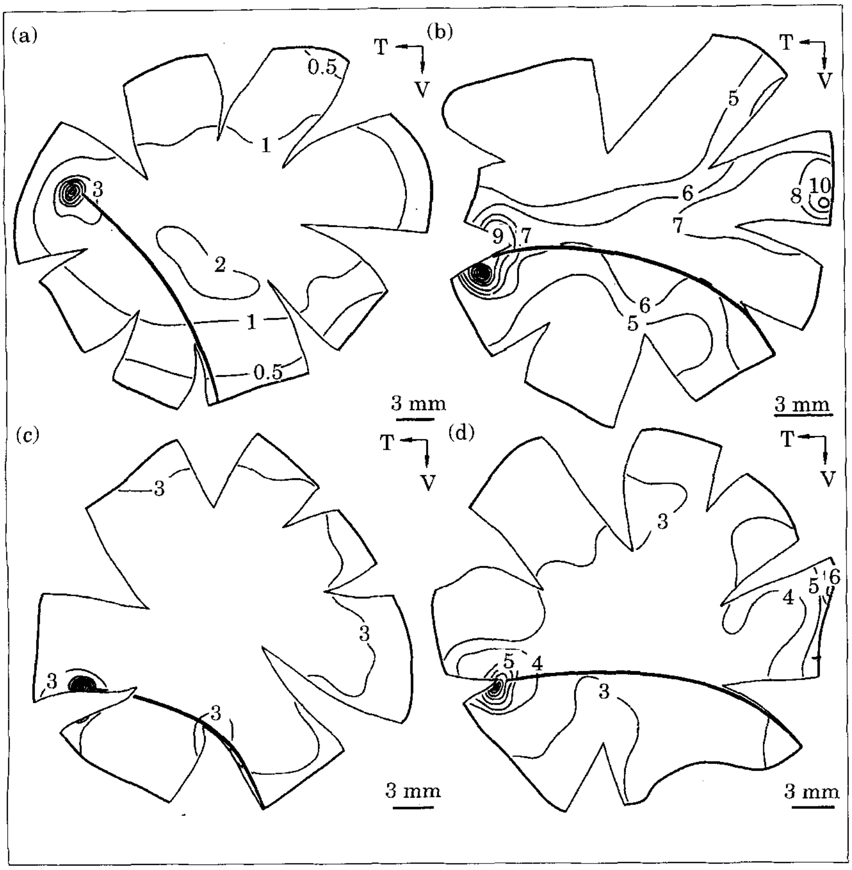

FIG. 5. Iso-density contour maps of the right eyes of four engybenthic deep-sea species showing the topography of the retinal ganglion cell distribution. (a) *Conocara murrayi*. (b) *Alepocephalus rostratus*. (c) *Bathytroctes microlepis*. (d) *Rouleina attrita*. All densities are $\times 10^3$ cells mm^{-2} and the peak density in (a)–(d) are 10 000, 24 000, 30 000 and 26 000 cells mm^{-2}, respectively. The peak densities of the nasal area centralis in (b) and (d) are 12 000 and 7000 cells mm^{-2}, respectively. The optic nerve head and falciform processes are depicted in black. T, temporal; V, ventral.

1843 [formerly *Choerodon albigena* (DeVis)] and the harlequin tuskfish *Lienardella fasciata* (Günther, 1867) (Collin & Pettigrew, 1988*c*). In both studies, the retinal topography remained fundamentally unchanged with the inclusion of the displaced amacrine cell population.

In the tubular-eyed species *S. michealsarsi*, *A. sladeni* and *A. aculeatus*, high centro-peripheral gradients (up to 37 : 1) indicate that the centro-lateral area centralis in the main retina provides significantly increased sampling of an image within the large binocular field subtended by the two eyes. This increased sampling in the main retina was originally suggested by Brauer (1908) for *Argyropelecus hemigymnus* Cocco, 1829 ($62{\cdot}5 \times 10^3$ cells mm^{-2}) and *Evermanella indica* Brauer, 1906 ($40{\cdot}0 \times 10^3$ cells mm^{-2}) and by Munk (1966) for *E. indica* ($23{\cdot}6 \times 10^3$ cells mm^{-2}) and *Winteria telescopa* Brauer, 1901 ($17{\cdot}7 \times 10^3$ cells mm^{-2}). Unfortunately, these sampled densities were not measured systematically. Moreover, the topographic position of each areae

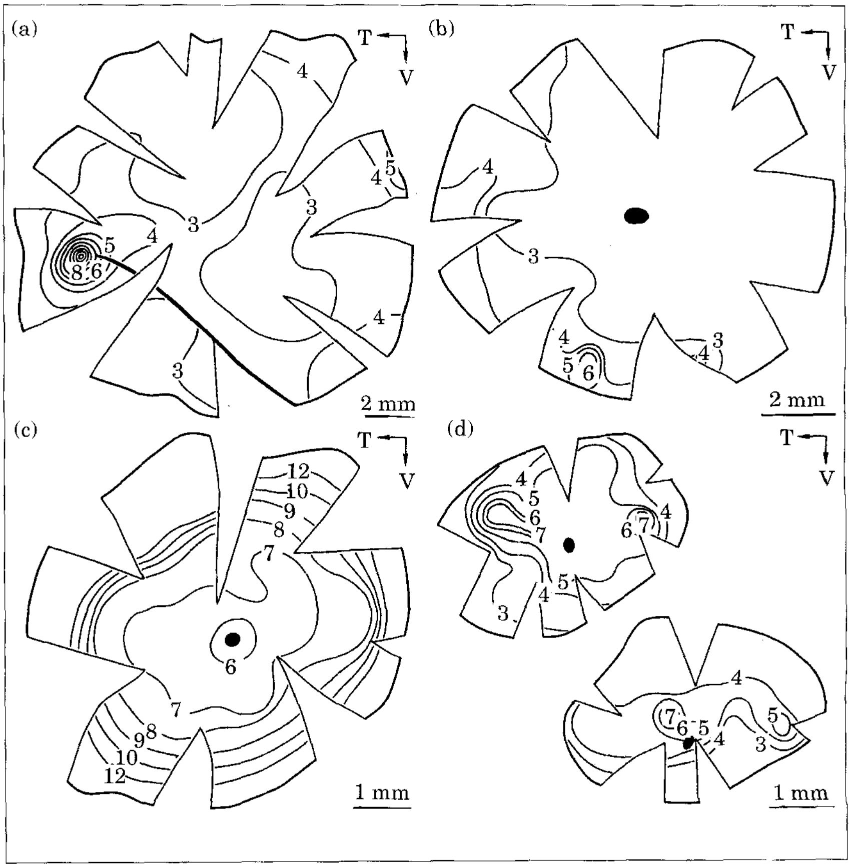

FIG. 6. Iso-density contour maps of the right eyes of three bathypelagic (a–c) and one benthic (d) deep-sea species showing the topography of the retinal ganglion cell distribution. (a) *Platytroctes apus*. (b) *Synaphobranchus kaupi*. (c) *Serrivomer beani*. (d) *Bathypterois dubius*. All densities are $\times 10^3$ cells mm^{-2} and the peak density in (a) is 26 000 cells mm^{-2}. The optic nerve head and falciform processes are depicted in black. T, temporal; V, ventral.

could not be identified definitively and retinal ganglion cell density was measured in sectioned material which may have produced retinal shrinkage and artifactual increases in cell density.

The finding of an acute zone in the tubular-eyed species of this study supports the premise that the main retina receives a focused image which has been postulated only theoretically by the fulfilment of Matthiessen's ratio (Matthiessen, 1880). Matthiessen's ratio states that the focal length of the fish lens is approximately 2·55 times the lens radius (ranging from 2·20 to 2·80, Matthiessen, 1880). This ratio was confirmed by cryosectioning the unfixed eyes of *A. aculeatus* (2·67) and *Scopelarchus* sp. (2·59) (N. J. Marshall & R. H. Douglas, pers. comm.) on board ship which negates the effects of fixation artefact produced when embedding and sectioning the eye in resin/wax. However, this procedure also revealed that the accessory retina is too close to the lens to provide a clear image and must, therefore, simply provide an extended field of view into lateral visual space presumably for unresolved light detection.

The centro-lateral position of the area centralis in the main retina of the tubular-eyed species provides a clue to the part of the visual field a prey item has to be located to be visualized with high acuity and captured successfully. Given that these species possess little or no eye mobility, the authors suggest that they do not fixate actively on prey items but either scan their environment by swimming actively in search of prey or are carried by the currents while maintaining neutral buoyancy and lie in wait for prey. In either case, an area centralis may signal the point within the caudo-medial region of the visual field at which to strike for prey.

A distinct group of mid-water (mesopelagic) teleosts possess a unique topographic distribution of cells within the retinal ganglion cell layer. *Myctophum punctatum*, *L. macdonaldi* and *S. beani* in addition to a number of other mesopelagic species i.e. *Lobianchia gemellari* (Cocco, 1838) and *Halargyreus johnsonii* Günther, 1862 (data not presented), all possess a concentric increase in cell density towards the retinal margin up to a peak of $19{\cdot}4 \times 10^3$ cells mm^{-2}. Since this arrangement is common to a number of species from different families, but all sharing a common habitat, a similar function is suggested. A peripheral increase in cell density may indicate a need for increased resolution in the peripheral field or may simply reflect a high degree of neurogenesis at the retinal margins. However, comparisons of the number of axons in the optic nerve (using electron microscopy) with the number of cells in the ganglion cell layer (using retrograde labelling and morphological analysis) in *L. macdonaldi* suggests that the high densities lying at the retinal margins are in fact displaced amacrine cells (S. P. Collin, R. V. Hoskins & J. C. Partridge, unpubl. data). When the distribution of the ganglion cells alone is examined in this species, their density is remarkably constant with a slight increase (gradient of less than 2 : 1) towards the central retina. Therefore, as in shallow-water species, the proportion of displaced amacrine cells is lowest in the area of highest ganglion cell density (Collin & Pettigrew, 1988*c*). However, this finding, if common to all the mesopelagic species with this type of density profile, means that peripheral vision is not increased and that the retinae of these species are not remarkably specialized for acute vision, relying on increased sensitivity (although summation ratios between the photoreceptors and the ganglion cells are still to be examined) and other sensory modalities.

The alepocephalids *C. murrayi*, *A. rostratus*, *B. microlepis*, *R. attrita* and *X. copei* all show marked increases in retinal ganglion cell density near the temporal margin. Temporo-peripheral gradients of up to 37 : 1 signify a sampling of an image in the frontal binocular field with high spatial resolving power (up to 13·5 cycles per degree in *B. microlepis*, Table I). A high density of tightly-packed slender rods in the perifoveal region also ensures a low summation ratio. In addition to the high sampling of an image in this region, the deep or convexiclivate fovea (also found in the two searsids examined) may also have an optical effect. The function of the fovea was considered in birds, lizards and some shallow-water fishes by Walls (1942), and magnification of the image was suggested to take place at the vitreous–retinal boundary, potentially increasing resolution. Pumphrey (1948) dismissed Walls' theory in favour of the theory that the slopes of the fovea distort the image, not magnify it, thereby causing the image of a moving object to jump as it crosses the fovea. This image distortion

provides a means of maintaining fixation of a point source of light by accentuating its movement since it will only appear symmetrical when it falls directly in the centre of the foveal pit. The finding of a deposit of radial fibres within the nerve fibre layer in the foveal depression of the alepocephalids and searsids found in this study (and others, Locket, 1971*b*, 1985, 1992) suggests a complex optical effect which may not be resolved until refractive index measurements are made of this densely staining region.

Like the alepocephalids, the searsids *S. koefoedi* and *P. apus* also possess large eyes with a wide pupil and a rostral aphakic space. The presence of a sighting groove, the rostral movement of the spherical lens during accommodation (Locket, 1971*b*) and a temporal fovea is suggestive of a visual predator using binocular vision. Once imaged on both foveae, prey position will be signalled within fine limits and accuracy will be enhanced by the signalling of image eccentricity. In addition, the direction of skew of a point source of light is suggested by Locket (1985) to act as a range-finding mechanism when the two foveal axes are coincident. However, the foveal axes may vary from the upper frontal visual field (e.g. *B. microlepis*) to the lower frontal visual field (e.g. *C. murrayi*) signified by the location of the fovea and the position of the eyes and region of maximum binocular overlap.

The deepwater bass *H. sherborni* possesses large eyes, a yellow tapetum and a fovea with multiple banks of rods (Locket, 1992). The presence of a fovea with a concomitant peak of $24{\cdot}5 \times 10^3$ ganglion cells mm^{-2} suggests that this species is a visual predator. However, although the spatial resolving power was not calculated for this species, summation ratios may be high, given the multiple banks of rods which lie sclerad of the fovea and the tight packing of the receptors (spaced about 2·8 μm apart, Locket, 1992).

The retina of the arrowtooth eel *Synaphobranchus kaupi* possesses a ventro-temporal area centralis of $6{\cdot}3 \times 10^3$ cells mm^{-2} (the lowest peak density of any of the species examined in this study) with a low ventro-peripheral density gradient. The position of the area centralis suggests that this species may utilize its dorso-frontal visual field for acute vision to prey on its staple diet of decapods and amphipods (Whitehead *et al.*, 1986). Since this species is known to descend to deeper areas during development (post metamorphosis, Bruun, 1937), and was retrieved with an OTSB (bottom trawling net), the 370 mm specimen of *S. kaupi* examined in this study could be regarded as an adult with a bathypelagic habitat. It is interesting to note that recent studies of the patterns of rod proliferation in this species reveal a non-linear drop in mitotic activity in specimens larger than 200 mm with a proliferation rate similar to that of surface-living fishes (Johns & Fernald, 1981; Fröhlich *et al.*, 1995). Therefore, visual sensitivity may also be maintained by the competing processes of rod proliferation throughout the retina, causing stretching of the retina during growth and the addition of other retinal neurons (including ganglion cells) at the retinal margins. Whether the increased mitotic activity of rod proliferation in the juveniles (up to a length of 200 mm, Fröhlich *et al.*, 1995) gives rise to increased stretching of the retina and therefore a different topographic ganglion cell distribution remains unknown.

Although some species of tripodfishes are thought to possess degenerate eyes (e.g. *Bathypterois longipes* Günther, 1878, Munk, 1965), *B. dubius* has two retinal

specializations in the form of a temporal and a nasal area centralis subtending the rostral and caudal visual fields, respectively. *Bathypterois dubius* sits on the bottom on specially strengthened rays of its pelvic and caudal fins feeding on tiny planktonic crustaceans, especially copepods. This species may utilize both areae when initially lying in wait perched towards a slight prey-carrying current (temporal area) with its long pectoral fin rays projected forward (Bruun, 1956) while signalling the approach of potential predators or other prey in its lateral visual field (nasal area).

The densities of retinal ganglion cells provided in this study reveal a range from $6{\cdot}3 \times 10^3$ cells mm^{-2} in *S. kaupi* to $50{\cdot}6 \times 10^3$ cells mm^{-2} in *A. aculeatus*. This range in density rivals that for shallow-water species which range from $6{\cdot}0 \times 10^3$ cells mm^{-2} in the frogfish *Halophryne diemensis* (LeSueur, 1824) (Collin & Pettigrew, 1988*a*) and the goldfish *Carassius auratus* (Linnaeus, 1758) (Mednick & Springer, 1988) to approximately $51{\cdot}0 \times 10^3$ cells mm^{-2} in the triggerfish *Balistoides conspicillum* (Bloch & Schneider, 1901) (Collin & Pettigrew, 1988*b*) and the red-throated emperor *Lethrinus miniatus* Schneider, 1801 (formerly *Lethrinus chrysostomus* Richardson) (Collin & Pettigrew, 1988*b*).

The spatial resolving power varies from 5·6 cycles per degree (10·7 min of arc) in *S. michaelsarsi* to 13·5 cycles per degree (4·4 min of arc) in *B. microlepis*. This translates to an ability to resolve two point sources of light (bioluminescent point sources) separated by 37·3 mm (*S. michaelsarsi*) and 15·4 mm (*B. microlepis*) at a distance of 6 m. By way of comparison, humans with a standard foveal visual acuity of 1 min of arc can resolve two lines separated by 1·75 mm at a distance of 6 m.

The finding that most of the species examined possess zones of increased ganglion cell density subtending a specific region of their visual field, suggests that deep-water teleosts, like shallow-water teleosts of a coral reef, rely on high spatial resolving power for prey localization and predator surveillance. The measurements for deep-sea species compare to a range of spatial resolving powers in the shallow-water species of 5·0 cycles per degree (12 min of arc) in the banded toado *Sphaeroides pleurostictus* (Günther, 1871) and 14 cycles per degree (4·3 min of arc) in the coral cod *Cephalopholis miniatus* (Forsskal, 1775) (Collin & Pettigrew, 1989), also calculated from retinal ganglion cell densities. The tuna *Euthynnus affinis* (Cantor, 1849) (Nakamura, 1968) and the planktivorous sunfish *Lepomis macrochirus* Rafinesque, 1819 (Hairston *et al.*, 1982) possess a behaviourally measured grating acuity of 8·1 cycles per degree (7·4 min of arc) and 4·3 cycles per degree (14 min of arc). However, there are a proportion of mesopelagic deep-water species that do not possess a remarkably specialized zone for acute vision (such as in the goldfish *Carassius auratus*, Mednick & Springer, 1988), relying on sensitivity (in conjunction with other sensory modalities) rather than high spatial resolving power.

Therefore, although the range of ecological habitats may not be as diverse in the darkness of the deep sea as it is in the well lit waters near the surface, most species in the deep sea still possess a comparable range (qualitatively and quantitatively) of visual capabilities and retinal specializations. The selective pressures upon the visual system to overcome the trade-off between retinal resolving power and sensitivity in an environment where little or no sunlight

penetrates must be enormous and future studies will undoubtedly reveal even more wonderful ocular specializations.

The authors thank Peter Herring, Nigel Merrett and the many scientists and crew of the RRS 'Challenger' and RRS 'Discovery' for their invitation and invaluable assistance in the collection and identification of the specimens collected; Ron Douglas for his generous efforts in providing and packing chemicals; Robert V. Hoskins, Darren Lloyd, Tom Walmsley, Simon Milledge and Kevin Lloyd for their technical assistance and patience in counting some of the species; and Ron Douglas, H.-Jochen Wagner and N. Justin Marshall for useful discussions. S. P. Collin is a QE II Research Fellow and this research was funded by the Australian Research Council, the British Council and the Ian Potter and George Alexander Foundations. J. C. Partridge is a Royal Society University Research Fellow and the research cruises were funded by the Natural Environmental Research Council (NERC) of the United Kingdom.

References

Ahlbert, I.-B. (1976). Organization of the cone cells in the retinae of salmon (*Salmo salar*) and trout (*Salmo trutta trutta*) in relation to their feeding habits. *Acta Zoologica (Stockholm)* **57,** 13–35.

Brauer, A. (1908). Die Tiefseefische. 2. Anatomische Teil. *Wissenschaftliche Ergebnisse Deutschen Tiefsee-Expedition auf dem Dampfer " Valdivia "* **15,** 1–266.

Bruun, A. F. (1937). Contributions to the life histories of deep-sea eels: Synaphobranchidae. *Dana Report* **9,** 1–31.

Bruun, A. F. (1956). Animal life of the deep-sea bottom. In *The Galathea Deep Sea Expedition 1950–1952* (Bruun, A. F., ed.), pp. 149–195. London: George Allen and Unwin.

Collin, S. P. (1989). The topography and morphology of retinal ganglion cells in the coral trout, *Plectropoma leopardus* (Serranidae): A retrograde cobaltous-lysine study. *Journal of Comparative Neurology* **281,** 143–158.

Collin, S. P. & Collin, H. B. (1988*a*). Topographic analysis of the retinal ganglion cell layer and the optic nerve in the sandlance, *Limnichthyes fasciatus* (Creeiidae, Perciformes). *Journal of Comparative Neurology* **278,** 226–241.

Collin, S. P. & Collin, H. B. (1988*b*). The morphology of the retina and lens of the sandlance *Limnichthyes fasciatus* (Creeiidae). *Experimental Biology* **47,** 208–218.

Collin, S. P. & Pettigrew, J. D. (1988*a*). Retinal topography in reef teleosts. I. Some species with well developed areae but poorly developed streaks. *Brain Behavior and Evolution* **31,** 269–282.

Collin, S. P. & Pettigrew, J. D. (1988*b*). Retinal topography in reef teleosts. II. Some species with prominent horizontal streaks and high density areae. *Brain Behavior and Evolution* **31,** 283–295.

Collin, S. P. & Pettigrew, J. D. (1988*c*). Retinal ganglion cell topography in teleosts: A comparison between Nissl-stained material and retrograde labelling from the optic nerve. *Journal of Comparative Neurology* **276,** 412–422.

Collin, S. P. & Pettigrew, J. D. (1989). Quantitative comparison of the limits on visual spatial resolution set by the ganglion cell layer in twelve species of reef teleosts. *Brain Behavior and Evolution* **34,** 184–192.

Collin, S. P., Lloyd, D. J. & Partridge, J. C. (1994). Retinal ganglion cell topography in deep-sea fishes: intrafamilial variation within the family alepocephalidae. *Proceedings of the Australian Neuroscience Society* **4,** 205.

Denton, E. J. (1990). Light and vision at depths greater than 200 metres. In *Light and Life at Sea* (Herring, P. J., Campbell, A. K., Whitfield, M. & Maddock, L., eds), pp. 127–148. Cambridge: Cambridge University Press.

Denton, E. J. & Locket, N. A. (1989). Possible wavelength discrimination by multibank retinae in deep-sea fishes. *Journal of the Marine Biological Association of the United Kingdom* **69,** 409–435.

Fernald, R. D. (1988). Aquatic adaptations in fish eyes. In *Sensory Biology of Aquatic Animals* (Atema, J., Fay, R. R., Popper, A. N. & Tavolga, W. N., eds), pp. 435–466. Berlin: Springer-Verlag.

Fröhlich, E., Negishi, K. & Wagner, H.-J. (1995). Patterns of rod proliferation in deep-sea fish retinae. *Vision Research* **35,** 1799–1811.

Hairston, Jr, N. G., Li, K. T. & Easter, Jr, S. S. (1982). Fish vision and the detection of planktivorous prey. *Science* **218,** 1240–1242.

Hughes, A. (1975). A quantitative analysis of the cat retinal ganglion cell topography. *Journal of Comparative Neurology* **163,** 107–128.

Hughes, A. (1977). The topography of vision in mammals. In *Handbook of Sensory Physiology*, Vol. 7 (Crescitelli, F., ed.), pp. 615–756. Berlin: Springer-Verlag.

Hughes, A. (1985). New perspectives in retinal organization. In *Progress in Retinal Research* (Osborne, N. N. & Chader, G., eds), pp. 243–313. New York: Pergamon Press.

Ito, H. & Murakami, T. (1984). Retinal ganglion cells in two teleost species, *Sebastiscus marmoratus* and *Navodon modestus. Journal of Comparative Neurology* **229,** 80–96.

Johns, P. R. & Fernald, R. D. (1981). Genesis of rods in teleost fish retina. *Nature (London)* **293,** 141–142.

Locket, N. A. (1971*a*). Retinal anatomy in some scopelarchid deep-sea fishes. *Proceedings of the Royal Society of London Series B* **178,** 161–184.

Locket, N. A. (1971*b*). Retinal structure in *Platytroctes apus*, a deep-sea fish with a pure rod fovea. *Journal of the Marine Biological Association of the United Kingdom* **51,** 79–91.

Locket, N. A. (1977). Adaptations to the deep-sea environment. In *The Visual System in Vertebrates. Handbook of Sensory Physiology*, Vol. VIII/5 (Crescitelli, F., ed.), pp. 67–192. New York: Springer-Verlag.

Locket, N. A. (1985). The multiple bank fovea of *Bajacalifornia drakei*, an alepocephalid deep-sea teleost. *Proceedings of the Royal Society of London Series B* **224,** 7–22.

Locket, N. A. (1992). Problems of deep foveas. *Australian and New Zealand Journal of Ophthalmology* **20,** 281–295.

Matthiessen, L. (1880). Untersuchungen über dem Aplanatismus und die Periscopie der Kristallinsen in den Augen der Fische. *Pflügers Archiv für die gesamte Physiologie des Menschen und der Tiere (Bonn)* **21,** 287–307.

Mednick, A. S. & Springer, A. D. (1988). Asymmetric distribution of retinal ganglion cells in goldfish. *Journal of Comparative Neurology* **268,** 49–59.

Munk, O. (1964). The eyes of some ceratioid fishes. *Dana Report* **61,** 1–16.

Munk, O. (1965). Ocular degeneration in deep-sea fishes. *Galathea Report* **8,** 21–31.

Munk, O. (1966*a*). On the retina of *Diretmus argenteus* Johnson 1863 (Diretmidae, Pisces). *Videnskabelige Meddelelser fra Dansk Naturhistorik Forening i Kjobenhavn* **129,** 73–80.

Munk, O. (1966*b*). Ocular anatomy of some deep-sea teleosts. *Dana Report* **70,** 1–62.

Munk, O. (1968). On the eye and so-called preorbital light organ of the isospondylous deep-sea fish *Bathylaco nigicans* Goode and Bean, 1896. *Galathea Report* **9,** 211–218.

Munk, O. (1970). On the occurrence and significance of horizontal band-shaped areae in teleosts. *Videnskabelige Meddelelser fra Dansk Naturhistorik Forening i Kjobenhavn* **133,** 85–120.

Nakamura, E. L. (1968). Visual acuity of two tunas, *Kasuwonus pelamis* and *Euthynnis affinis. Copeia* **1,** 41–49.

O'Connell, C. P. (1963). The structure of the eye of *Sardinops caerulea, Engraulis mordax*, and four other pelagic marine teleosts. *Journal of Morphology* **113,** 287–330.

Pettigrew, J. D., Dreher, B., Hopkins, C. S., McCall, M. J. & Brown, M. (1988). Peak density and distribution of ganglion cells in the retinae of microchiropteran bats: Implications for visual acuity. *Brain Behaviour and Evolution* **32,** 39–56.

Pietsch, T. W. (1978). The feeding mechanism of *Stylephorus chordatus* (Teleostei: Lampridiformes): Functional and ecological implications. *Copeia* **1978,** 255–262.

Pumphrey, R. J. (1948). The theory of the fovea. *Journal of Experimental Biology* **25,** 299–312.

Reymond, L. (1985). Spatial visual acuity of the eagle, *Aguila audax*: A behavioural, optical and anatomical investigation. *Vision Research* **25,** 1477–1491.

Schwassmann, H. O. (1968). Visual projection upon the optic tectum in foveate marine teleosts. *Vision Research* **8,** 1337–1348.

Vilter, V. (1954). Différenciation fovéale dans l'appareil visuel d'un poisson abyssal, le *Bathylagus benedicti. Comptes Rendu des Séances de la Société Biologie* **148,** 59–63.

Walls, G. L. (1942). *The Vertebrate Eye and its Adaptive Radiation.* London: Cranbrook Institute of Science Bulletin.

Whitehead, P. J. P., Bauchot, M.-L, Hureau J.-C., Nielsen, J. & Tortonese, E. (1986). *Fishes of the North-eastern Atlantic and the Mediterranean,* Vols I–III. Paris: UNESCO.

Zaunreiter, M., Junger, H. & Kotrschal, K. (1991). Retinal structure; physiology and pharmacology. Retinal morphology of cyprinid fishes; a quantitative histological study of ontogenetic changes and interspecific variation. *Vision Research* **31,** 383–394.
